# Longitudinal and cross-sectional associations of myocardial stress markers with kidney function and chronic kidney disease in the BiomarCaRE project

**DOI:** 10.1038/s41598-026-37377-2

**Published:** 2026-02-23

**Authors:** Jie-sheng Lin, Tanja Zeller, Wolfgang Koenig, Pekka Jousilahti, Frank Kee, Licia Iacoviello, Hugh Tunstall-Pedoe, Stefan Söderberg, Giancarlo Cesana, Luigi Palmieri, Veikko Salomaa, Julia de Man Lapidoth, Roberto De Ponti, Chiara Donfrancesco, Thiess Lorenz, Kari Kuulasmaa, Stefan Blankenberg, Annette Peters, Barbara Thorand

**Affiliations:** 1 Institute of Epidemiology, Helmholtz Zentrum München, German Research Center for Environmental Health (GmbH), Neuherberg, Germany; 2 Institute for Medical Information Processing, Biometry, and Epidemiology (IBE), Faculty of Medicine, LMU Munich, Pettenkofer School of Public Health, Munich, Germany; 3 School of Public Health and Emergency Management, School of Medicine, Southern University of Science and Technology, Shenzhen, China; 4 Institute for Cardiogenetics, University of Lübeck, University Hospital Schleswig-Holstein, Lübeck, Germany; 5 German Center for Cardiovascular Research (DZHK), Partner Site Hamburg/Kiel/Lübeck, Lübeck, Germany; 6 German Heart Center, Munich, Technical University of Munich, Munich, Germany; 7 German Center for Cardiovascular Research (DZHK), Partner Site Munich Heart Alliance, Munich, Germany; 8 Institute of Epidemiology and Medical Biometry, University of Ulm, Ulm, Germany; 9 Department of Public Health, Finnish Institute for Health and Welfare (THL), Helsinki, Finland; 10 Centre for Public Health, Queen’s University Belfast, Belfast, Northern Ireland UK; 11 Department of Epidemiology and Prevention, IRCCS Neuromed, Pozzilli, Italy; 12 Department of Medicine and Surgery, LUM University, Casamassima, Italy; 13 Cardiovascular Epidemiology Unit, Institute of Cardiovascular Research, University of Dundee, Dundee, Scotland; 14 Department of Public Health and Clinical Medicine, Umeå University, Umeå, Sweden; 15 Department of Medicine and Surgery, University of Milano-Bicocca, Milan, Italy; 16 Department of Cardiovascular, Endocrine-Metabolic Diseases and Aging, Istituto Superiore Di Sanità-ISS, Rome, Italy; 17 Research Center in Epidemiology and Preventive Medicine (EPIMED), Department of Medicine and Surgery, University of Insubria, Varese, Italy; 18 Center for Population Health Innovation (POINT), University Heart and Vascular Center Hamburg, University Medical Center Hamburg-Eppendorf, Hamburg, Germany; 19 Department of Cardiology, University Heart and Vascular Center Hamburg, University Medical Center Hamburg-Eppendorf, Hamburg, Germany; 20 German Center for Diabetes Research (DZD), Partner München-Neuherberg, Neuherberg, Germany; 21 Department of Public Health, University of Helsinki, Helsinki, Finland; 22 Department of Internal Medicine, University of Turku, Turku, Finland

**Keywords:** MR-proADM, MR-proANP, NT-p, roBNP, Kidney function, Chronic kidney disease, Epidemiology, Biomarkers, Kidney diseases

## Abstract

Given the complex relationship between cardiovascular disease (CVD) and chronic kidney disease (CKD), CVD-related markers may serve as CKD biomarkers. We examined associations of three major CVD-markers [mid-regional pro-adrenomedullin (MR-proADM), MR-pro-atrial natriuretic peptide (MR-proANP), and N-terminal pro-B-type natriuretic peptide (NT-proBNP)] with CKD. Cross-sectional analyses included up to 61,830 participants, and longitudinal analyses (NT-proBNP only) 4205 individuals. Kidney function was assessed by estimated glomerular filtration rate (eGFR) using creatinine, cystatin C, or both (eGFRcr-cys). Markers were categorized into four groups. Cross-sectional analyses found that higher levels of all three markers were consistently associated with lower eGFR and higher CKD prevalence. For example, per 1 standard deviation (SD) increase in log-transformed NT-proBNP, corresponding to a 2.71-fold increase in the original concentration, was associated with -2.35 (-2.49, -2.21) ml/min/1.73m^2^ lower eGFRcr-cys, and the highest NT-proBNP group had a 5.72-fold higher odds of CKDcr-cys (eGFRcr-cys < 60 ml/min/1.73m^2^) compared with the lowest. Associations with eGFR were stronger among participants with CVD and diabetes. In longitudinal analyses, participants with higher baseline NT-proBNP had faster declines in eGFR, with a 10-year decline of -1.37 (-1.77, -0.98) ml/min/1.73m^2^ eGFRcr-cys per 1 SD increase, and higher CKD incidence. These findings suggest MR-proADM, MR-proANP, and NT-proBNP as CKD biomarkers.

**Supplementary Information:**

The online version contains supplementary material available at 10.1038/s41598-026-37377-2.

## Introduction

Chronic kidney disease (CKD) is characterized by a gradual kidney function decline, with a median prevalence of 9.5% among adults and children worldwide^1^. Given the asymptomatic onset, early CKD detection remains challenging. Heart and kidneys interact bidirectionally, and a spectrum of disorders involving both organs is termed cardiorenal syndrome^2^. CKD and cardiovascular disease (CVD) share common risk factors such as diabetes^3^, and mechanisms of disease development such as overactivation of the renin–angiotensin–aldosterone system (RAAS)^4,5^. Given their complex interactions, mid-regional pro-adrenomedullin (MR-proADM), MR-pro-atrial natriuretic peptide (MR-proANP), and N-terminal pro-B-type natriuretic peptide (NT-proBNP), some of the prominent CVD-related markers^6–9^, may be useful as CKD biomarkers.

MR-proADM, MR-proANP, and NT-proBNP are stable surrogate markers for ADM, ANP, and BNP, respectively, because the latter three markers have shorter half-lives. ANP, BNP, and ADM are all vasodilators. The elevations of these markers in the circulation usually occur in response to ventricular/atrial wall stretch and volume overload^7,8,10^, which in turn, are positively associated with impaired kidney function^3,11^. On the other hand, these markers are partially dependent on kidney clearance for elimination, and thus, the elevations of these markers are related to kidney function decline^12–15^. The above evidence suggests that these markers could be biomarkers for kidney function decline.

Epidemiological studies have shown that MR-proADM/ADM^16–18^, MR-proANP/ANP^16,19^, and NT-proBNP/BNP^20–31^, are positively associated with kidney function decline as well as the development and progression of CKD. Our previous study, including 233 proteomic biomarkers, also found that higher levels of ADM and NT-proBNP were associated with a faster kidney function decline and higher CKD incidence^32^. However, there are only a few studies regarding MR-proADM/ADM and MR-proANP/ANP, which have mainly focused on end-stage kidney disease (ESKD), the last stage of CKD, among patients with diabetes and CKD^16–18,33^, while studies in the general population are scarce^19^. Given diabetes is a major cause of CKD, the associations may differ between patients with diabetes and the general population, limiting the generalizability of previous findings. To date, the largest study investigating NT-proBNP with incident ESKD comprised 10,749 white and black participants, but they focused on ESKD and found the association differed by ethnicity^23^. Thus, large studies based on the general population with diverse genetic backgrounds are needed to further understand the associations of these markers with early stages of CKD, which is a more prevalent condition than ESKD.

We aimed to investigate the longitudinal (NT-proBNP only) and cross-sectional associations of MR-proADM, MR-proANP, and NT-proBNP with kidney function and CKD in a large general population from the Biomarkers for Cardiovascular Risk Assessment in Europe (BiomarCaRE) project^34^. Given they are CVD-related markers and both CVD and diabetes are major CKD causes, we further aimed to assess if the associations differed by CVD and diabetes.

## Methods

### Study design and population

BiomarCaRE has relied on the European population of the Monitoring of Trends and Determinants in Cardiovascular Diseases (MONICA), Risk, Genetics, Archiving and Monograph (MORGAM) project^35^. All studies included in BiomarCaRE were conducted in accordance with relevant guidelines and regulations, including the Declaration of Helsinki, and were approved by their respective local ethical committees. The names of the approving committees and approval numbers for each included study are provided in Table S1. For example, the FINRISK Study was approved by the National Public Health Institute of Finland (approval number 38/96). Written informed consent was obtained from all participants. We included seven studies from BiomarCaRE comprising 61,830 participants with data on both NT-proBNP and kidney function: FINRISK Study, MONICA/Cooperative Health Research in the Region of Augsburg (MONICA/KORA), Moli-sani Study, MONICA Brianza Study, Northern Sweden MONICA Study, Prospective Epidemiological Study of Myocardial Infarction Belfast (PRIME/Belfast), and Scottish Heart Health Extended Cohort (SHHEC). Short descriptions of each study are presented in Table S1. Data on MR-proANP (N = 9499) and MR-proADM (N = 9327) were only available in the FINRISK and PRIME/Belfast studies. Harmonized data on age, sex, body mass index, smoking status (current smoker), alcohol consumption, systolic blood pressure, use of antihypertensive medication, high-density lipoprotein cholesterol, low-density lipoprotein cholesterol, triglycerides, history of diabetes, and history of CVD were included. Diabetes (any type of diabetes) and CVD (myocardial ischemia, stroke, and, for some participants in the MONICA Brianza Study, angina pectoris) were assessed based on documented or self-reported history of these conditions. The categories of categorical variables are presented in Table 1. Missing values of covariates, up to 5.7%, were imputed by multiple imputation. For longitudinal analysis, follow-up measurement of kidney function was available in the MONICA/KORA study. Figure S1 shows that a maximum of 4205 participants with 10,208 observations were included in the longitudinal analysis, with a mean follow-up time of 11.1 ± 2.49 years.

**Table 1:** Baseline characteristics of participants included in the cross-sectional analysis across categories of NT-proBNP.

Characteristics	Categories of NT-proBNP^a^					***P***-value^b^
	Total (N = 61,830)	G1 (N = 30,939)	G2 (N = 21,284)	G3 (N = 7109)	G4 (N = 2498)	
	Mean (standard deviation) or number (%)					
Study cohort, N (%)						< 0.001
FINRISK	6858 (11.1)	3468 (11.2)	2359 (11.1)	775 (10.9)	256 (10.2)	
MONICA/KORA	5790 (9.4)	2834 (9.2)	2081 (9.8)	661 (9.3)	214 (8.6)	
Moli-sani	22,243 (36.0)	10,672 (34.5)	7764 (36.5)	2753 (38.7)	1054 (42.2)	
MONICA_Brianza	3623 (5.9)	2136 (6.9)	1143 (5.4)	281 (4.0)	63 (2.5)	
Northern Sweden MONICA	10,414 (16.8)	5560 (18.0)	3435 (16.1)	1026 (14.4)	393 (15.7)	
PRIME/Belfast	1539 (2.5)	1010 (3.3)	403 (1.9)	82 (1.2)	44 (1.8)	
SHHEC	11,363 (18.4)	5259 (17.0)	4099 (19.3)	1531 (21.5)	474 (19.0)	
Age (years)	51.8 (12.5)	47.7 (10.9)	53.1 (12.0)	60.2 (12.1)	66.3 (10.7)	< 0.001
Sex, N (%) female	32,209 (52.1)	12,716 (41.1)	13,755 (64.6)	4574 (64.3)	1164 (46.6)	< 0.001
Body mass index (kg/m^2^)	27.2 (6.07)	27.1 (5.74)	27.0 (6.24)	27.7 (6.29)	28.6 (7.46)	< 0.001
Current smoker, N (%)	17,229 (27.9)	9424 (30.5)	5631 (26.5)	1637 (23.0)	537 (21.5)	< 0.001
Alcohol consumption, N (%)						< 0.001
No alcohol consumption	20,265 (32.8)	9160 (29.6)	7546 (35.5)	2670 (37.6)	889 (35.6)	
> 0 and < 20 g/day	27,001 (43.7)	13,646 (44.1)	9324 (43.8)	3014 (42.4)	1017 (40.7)	
≥ 20 g/day	14,564 (23.6)	8133 (26.3)	4414 (20.7)	1425 (20.0)	592 (23.7)	
Systolic blood pressure (mm Hg)	135.8 (32.5)	132.3 (32.5)	136.0 (32.0)	144.3 (28.0)	152.1 (39.1)	< 0.001
Use of antihypertensive medication, N (%)	11,096 (17.9)	3420 (11.1)	4076 (19.2)	2354 (33.1)	1246 (49.9)	< 0.001
Hypertension, N (%)	27,574 (44.6)	11,352 (36.7)	9804 (46.1)	4485 (63.1)	1933 (77.4)	< 0.001
HDL-cholesterol (mmol/L)	1.46 (0.39)	1.41 (0.37)	1.51 (0.40)	1.51 (0.42)	1.41 (0.41)	< 0.001
LDL-cholesterol (mmol/L)	3.37 (1.02)	3.39 (1.00)	3.37 (1.02)	3.35 (1.05)	3.31 (1.09)	< 0.001
Triglycerides (mmol/L), median [IQR]	1.26 [0.91, 1.78]	1.28 [0.91, 1.83]	1.21 [0.89, 1.70]	1.29 [0.95, 1.80]	1.38 [1.03, 1.87]	< 0.001
Diabetes, N (%)	2890 (4.7)	1040 (3.4)	955 (4.5)	538 (7.6)	357 (14.3)	< 0.001
Cardiovascular diseases, N (%)	2563 (4.1)	528 (1.7)	730 (3.4)	708 (10.0)	597 (23.9)	< 0.001
eGFRcr (ml/min/1.73 m^2^)	97.3 (16.7)	100.8 (15.6)	96.4 (15.7)	90.3 (16.8)	80.7 (20.4)	< 0.001
CKDcr, N (%)	1833 (3.0)	539 (1.7)	543 (2.6)	364 (5.1)	387 (15.5)	< 0.001
eGFRcys (ml/min/1.73 m^2^)	94.4 (22.5)	99.5 (20.1)	93.3 (21.6)	84.0 (24.4)	71.3 (26.4)	< 0.001
CKDcys, N (%)	4663 (7.5)	878 (2.8)	1562 (7.3)	1310 (18.4)	913 (36.5)	< 0.001
eGFRcr-cys (ml/min/1.73 m^2^)	99.1 (19.4)	103.5 (17.4)	98.2 (18.4)	90.2 (20.6)	78.2 (23.6)	< 0.001
CKDcr-cys, N (%)	1956 (3.2)	365 (1.2)	530 (2.5)	538 (7.6)	523 (20.9)	< 0.001
MR-proADM, (nmol/l), median [IQR]^c^	0.47 [0.39, 0.56]	0.44 [0.37, 0.51]	0.48 [0.41, 0.58]	0.55 [0.47, 0.66]	0.65 [0.56, 0.78]	< 0.001
MR-proANP, (pmol/l), median [IQR]^c^	48.1 [35.2, 67.6]	39.6 [30.5, 52.6]	53.9 [41.7, 70.8]	78.9 [59.3, 103.0]	127.5 [91.4, 183.5]	< 0.001
NT-proBNP, (pg/ml), median [IQR]	48.0 [25.4, 89.6]	25.4 [15.9, 35.6]	72.5 [58.9, 92.1]	169.4 [142.6, 209.3]	501.2 [367.7, 837.2]	< 0.001

^a^Categories of NT-proBNP: G1: < 48; G2: 48–125; G3: 125–300; G4: ≥ 300 pg/ml.

^b^P-value was estimated by ANOVA test/Kruskal–Wallis test (continuous variables), or chi-squared test (categorical variables).

^c^MR-proADM and MR-proANP are only available in the FINRISK and PRIME/Belfast studies.

CKD, chronic kidney disease; cr, creatinine-based; cys, cystatin C-based; cr-cys, combined creatinine and cystatin C-based; eGFR, estimated glomerular filtration rate; G, group; HDL, high-density lipoprotein; IQR, interquartile range; LDL, low-density lipoprotein; MR-proADM, mid-regional pro-adrenomedullin; MR-proANP, mid-regional pro-atrial natriuretic peptide; NT-proBNP, N-terminal pro-B-type natriuretic peptide.

### Laboratory measurements

Details are presented in Text S1. Briefly, plasma MR-proADM and MR-proANP were measured using immunoluminometric assays. Serum NT-proBNP was measured using an electrochemiluminescence immunoassay. Plasma/serum creatinine was measured by the kinetic alkaline picrate Jaffe method or the enzymatic method. Serum cystatin C was measured using a Latex immunoassay. Table S2 presents intra- and inter-assay coefficients of variation.

### Assessment of kidney function and CKD

Kidney function was assessed by estimated glomerular filtration rate (eGFR), with creatinine-based (eGFRcr), cystatin C-based eGFR (eGFRcys), and combined creatinine and cystatin C-based eGFR (eGFRcr-cys), calculated using the Chronic Kidney Disease Epidemiology Collaboration equations (equations are presented in Text S1)^36,37^. CKD cases were defined as eGFR < 60 ml/min per 1.73m^2,38^. Incident CKD cases were defined as participants free of CKD at baseline and identified as CKD cases at any stage of the follow-up.

### Statistical analysis

All analyses were conducted by R v. 4.3.2 and RStudio v. 2023.12.1.

### Transformation of markers

A log10 transformation was applied to each marker, followed by Z-score standardization for comparability across different markers. The standard deviations (SD) of log-transformed markers were 0.43 for NT-proBNP, 0.22 for MR-proANP, and 0.12 for MR-proADM, corresponding approximately to 2.71-, 1.66-, and 1.32-fold increases in their original scale, respectively. Original levels of NT-proBNP were also categorized into four groups (G1, G2, G3, and G4): < 48, 48–125 (including 48), 125–300 (including 125), and ≥ 300 pg/ml. The value of 48 is the median value of NT-proBNP among the 61,830 participants. The values of 125 and 300 are used as thresholds to rule in heart failure (HF) and acute HF, respectively^6^. Categories of MR-proANP were: < 40, 40–80, 80–120, and ≥ 120 pmol/l. The value of 40 can be used as a threshold to rule out HF, while 120 is used for ruling in acute HF^6^. These cut-off values of MR-proANP corresponded to the 37.3^th^, 84.5^th^ (same as value 125 of NT-proBNP among 61,830 participants), and 95.6^th^ percentiles in the participants from the FINRISK and PRIME/Belfast studies. MR-proADM levels were categorized based on cut-off values corresponding to these percentiles: 0.425, 0.609, and 0.766 nmol/l.

### Associations with kidney function and CKD

In cross-sectional analysis, linear regression was used to estimate unstandardized β coefficients and 95% confidence intervals (CIs) of eGFR (ml/min/1.73m^2^) per 1 SD increase in log-transformed markers. Model 1: adjusted for age, sex, and study cohort. Model 2: model 1 plus body mass index, smoking status, alcohol consumption, systolic blood pressure, use of antihypertensive medication, high-density lipoprotein-cholesterol, log-transformed triglycerides, history of diabetes, and history of CVD. The same linear regression models were applied for grouped markers (G1-4), with G1 serving as the reference group. Logistic regression was used to estimate odds ratios (ORs) and 95% CIs for the association between biomarker levels (both log_10_-transformed and grouped) and prevalent CKD. In sensitivity analyses, CKD cases were redefined as all three eGFR < 60 ml/min per 1.73m^2^ and non-cases as all three eGFR ≥ 60 ml/min per 1.73m^2^.

In longitudinal analysis in the MONICA/KORA study, linear mixed-effects models were used to investigate the associations of NT-proBNP with change in eGFR, using “lme4” package. The follow-up duration was used as the timescale and divided by 10 to give a 10-year change. The fixed effects included standardized log-transformed NT-proBNP, follow-up duration, and their interaction terms, while random effects included random intercept and random slope (i.e., individual differences in eGFR change). The β coefficient of the interaction term is the impact of NT-proBNP on the 10-year change in eGFR. The same models were applied for grouped NT-proBNP (G1-4), with G1 serving as the reference group. Participants with at least one follow-up measurement on eGFR were included. For associations with incident CKD, interval-censored Cox regression models with 1000 bootstrap samples for 95% CIs estimation were performed, using “icenReg” package. The first two models in longitudinal analyses were similar to the cross-sectional analysis, with the exclusion of study cohort, and model 3 further adjusted for baseline eGFR. Since model 3 may be overcorrected^39^, we used model 2 as our main model. Linear mixed-effects models included time-varying covariates, except for sex. To address bias due to participants lost to follow-up (Figure S1), inverse probability weighting-weights were applied in all longitudinal analyses (details in Text S2).

### Stratified and sensitivity analyses

Interaction terms of standardized log-transformed markers with CVD or diabetes were included in the aforementioned final models in both cross-sectional and longitudinal analyses, and stratified analyses were performed if a significant interaction (p < 0.05) was found. Sensitivity analyses were conducted by further adjusting for low-density lipoprotein cholesterol, and E-values^40^ were calculated to assess the robustness of observed associations to potential unmeasured or uncontrolled confounders.

### Non-linear associations analysis

Restricted cubic spline functions with three knots were used to investigate the non-linear associations of original levels of three markers with prevalent CKDcr-cys in logistic regression models and non-linear associations of NT-proBNP with incident CKD in Cox proportional hazards models, adjusting for the covariates as in the aforementioned final models, using “rms” package. Participants with levels of markers < 2.5^th^ percentile or > 97.5^th^ percentile were excluded. P-values for nonlinearity were calculated using ANOVA tests.

## Results

The 61,830 participants included in the cross-sectional analysis had an average age of 51.8 ± 12.5 years (Table 1), and characteristics of participants across study cohorts are presented in Table S3 & S4. The baseline characteristics of participants from the MONICA/KORA study included in the longitudinal analysis are shown in Table S5.

### Cross-sectional associations

**Fig. 1:**
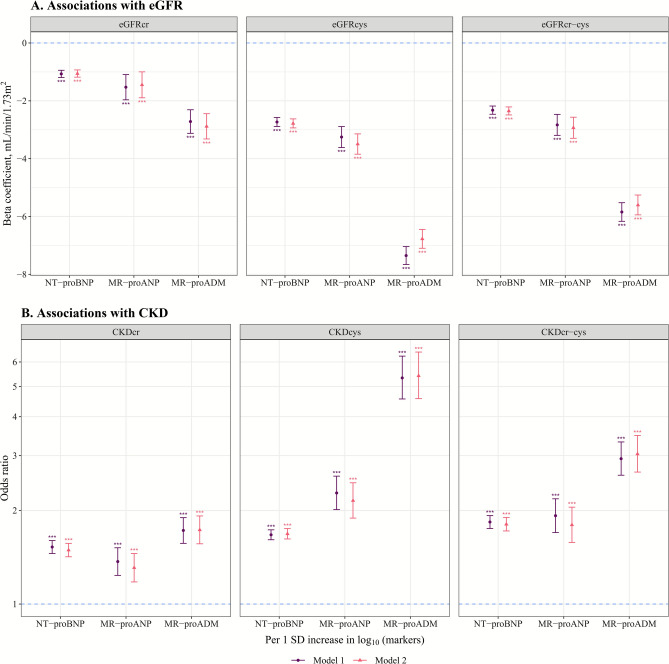
Cross-sectional associations of 3 myocardial stress markers with kidney function and CKD. (**A**). Linear regression was used to estimate beta coefficients and 95% CI of eGFR per 1 SD increase in log-transformed markers. (**B**). Logistic regression was used to estimate odds ratios of CKD. Detailed information on adjusted models is described in Table 2 & 3. Data from 61,830 participants for NT-proBNP, 9499 for MR-proANP, and 9327 for MR-proADM were included in these analyses. CI, confidence interval; CKD, chronic kidney disease; cr, creatinine-based; cys, cystatin C-based; cr-cys, combined creatinine and cystatin C-based; eGFR, estimated glomerular filtration rate; MR-proADM, mid-regional pro-adrenomedullin; MR-proANP, mid-regional pro-atrial natriuretic peptide; NT-proBNP, N-terminal pro-B-type natriuretic peptide; SD, standard deviation; ^*^ p < 0.05, ^**^ p < 0.01, ^***^ p < 0.001.

Higher levels of three myocardial stress markers were associated with lower eGFR across all three eGFR assessments (Table S6 & Fig. 1A). In model 2, the β (95% CIs) of eGFRcr-cys per 1 SD increase in log-transformed markers were −2.35 (−2.49, −2.21) ml/min/1.73m^2^ for NT-proBNP, −2.93 (−3.30, −2.57) for MR-proANP, and −5.60 (−5.94, −5.26) for MR-proADM, respectively (Table 2). A 1 SD increase on the log₁₀ scale corresponds approximately to 2.71-, 1.66-, and 1.32-fold increases in the original concentrations. Figure S2 shows that compared with G1, G2-G4 with higher levels of markers had significantly lower eGFR. For instance, β (95% CIs) of eGFRcr-cys for G2-G4 compared with G1 of NT-proBNP in model 2 were −1.41 (−1.69, −1.14), −4.10 (−4.52, −3.68), and −11.2 (−11.8, −10.5), respectively (Table 2).

**Table 2:** Cross-sectional associations of 3 myocardial stress markers with kidney function assessed by eGFRcr-cys^a^.

Markers	Items	Categories of markers^b^				per 1 SD increase in log-transformed
		**G1**	**G2**	**G3**	**G4**	
NT-proBNP	Median, pg/ml	25.4	72.5	169.4	501.2	48.0
	N	30,939	21,284	7109	2498	61,830
	Model 1	Ref	−1.22 (−1.51, −0.94) ***	−4.23 (−4.66, −3.80) ***	−11.7 (−12.4, −11.0) ***	−2.32 (−2.47, −2.18) ***
	Model 2	Ref	−1.41 (−1.69, −1.14) ***	−4.10 (−4.52, −3.68) ***	−11.2 (−11.8, −10.5) ***	−2.35 (−2.49, −2.21) ***
MR-proANP	Median, pmol/l	30.8	53.7	92.8	147.2	46.8
	N	3540	4493	1048	418	9499
	Model 1	Ref	−2.16 (−2.88, −1.45) ***	−5.30 (−6.43, −4.17) ***	−12.4 (−13.9, −10.8) ***	−2.83 (−3.20, −2.47) ***
	Model 2	Ref	−2.68 (−3.39, −1.98) ***	−5.74 (−6.86, −4.62) ***	−11.8 (−13.4, −10.2) ***	−2.93 (−3.30, −2.57) ***
MR-proADM	Median, nmol/l	0.37	0.50	0.66	0.86	0.46
	N	3492	4397	1029	409	9327
	Model 1	Ref	−5.55 (−6.20, −4.89) ***	−12.4 (−13.5, −11.4) ***	−22.2 (−23.7, −20.7) ***	−5.84 (−6.17, −5.52) ***
	Model 2	Ref	−5.24 (−5.90, −4.58) ***	−11.7 (−12.7, −10.6) ***	−21.0 (−22.6, −19.5) ***	−5.60 (−5.94, −5.26) ***

^a^Linear regression was used to estimate beta coefficients and 95% CI of eGFR for G2-4 compared with G1 of markers, as well as for per 1 SD increase in log-transformed markers. Only results for eGFRcr-cys are presented in this table, while results for all three eGFR assessments are presented in Table S6.

^b^Categories of NT-proBNP: G1: < 48; G2: 48–125; G3: 125–300; G4: ≥ 300 pg/ml. Categories of MR-proANP: G1: < 40; G2: 40–80; G3: 80–120; G4: ≥ 120 pmol/l. Categories of MR-proADM: G1: < 0.425; G2: 0.425–0.609; G3: 0.609–0.766; G4: ≥ 0.766 nmol/l.

Model 1: adjusted for age, sex, and study cohort;.

Model 2: model 1 plus body mass index, smoking status, alcohol consumption, systolic blood pressure, use of antihypertensive medication, high-density lipoprotein cholesterol, log-transformed triglycerides, diabetes, and cardiovascular diseases.

CI, confidence interval; G, group; eGFR, estimated glomerular filtration rate; eGFRcr-cys, creatinine and cystatin C-based eGFR; MR-proADM, mid-regional pro-adrenomedullin; MR-proANP, mid-regional pro-atrial natriuretic peptide; NT-proBNP, N-terminal pro-B-type natriuretic peptide; Ref, reference; SD, standard deviation.

* p < 0.05, ** p < 0.01, *** p < 0.001.

Results for CKD showed consistent associations, with higher levels of three markers associated with a higher prevalence of CKD across all three CKD assessments (Table S7 & Fig. 1B). Fully-adjusted ORs (95% CIs) of CKDcr-cys per 1 SD increase in log-transformed markers were, 1.81 (1.72, 1.90) for NT-proBNP, 1.80 (1.58, 2.05) for MR-proANP, and 3.04 (2.65, 3.48) for MR-proADM, respectively (Table 3). Associations with categories of markers also revealed significantly higher CKD prevalence in G2-G4 compared with G1, with G4 demonstrating the strongest associations (Figure S3). For example, Table 3 shows that fully-adjusted ORs (95% CIs) of CKDcr-cys across G2-G4 compared with G1 of NT-proBNP were 1.37 (1.19, 1.58), 2.56 (2.19, 2.99), and 5.72 (4.82, 6.78). In sensitivity analyses using redefined CKD cases, the association with per 1 SD increase in log-transformed markers remained significant with higher ORs but wider 95% CIs (Table S8 & Figure S4).

**Table 3:** Cross-sectional associations of 3 myocardial stress markers with CKDcr-cys ^a^.

Markers	Items	Categories of markers^b^				per 1 SD increase in log-transformed
		**G1**	**G2**	**G3**	**G4**	
NT-proBNP	Median, pg/ml	25.4	72.5	169.4	501.2	48.0
	Cases/controls (%)	365/30,574 (1.2%)	530/20,754 (2.6%)	538/6571 (8.2%)	523/1975 (26.5%)	1956/59,874 (3.3%)
	Model 1	Ref	1.35 (1.17, 1.55) ***	2.62 (2.25, 3.06) ***	6.14 (5.22, 7.24) ***	1.84 (1.75, 1.93) ***
	Model 2	Ref	1.37 (1.19, 1.58) ***	2.56 (2.19, 2.99) ***	5.72 (4.82, 6.78) ***	1.81 (1.72, 1.90) ***
MR-proANP	Median, pmol/l	30.8	53.7	92.8	147.2	46.8
	Cases/controls (%)	39/3501 (1.1%)	124/4369 (2.8%)	58/990 (5.9%)	79/339 (23.3%)	300/9199 (3.3%)
	Model 1	Ref	1.48 (1.00, 2.21)	2.22 (1.40, 3.57) ***	7.35 (4.63, 11.8) ***	1.92 (1.70, 2.18) ***
	Model 2	Ref	1.54 (1.05, 2.31) *	2.17 (1.36, 3.50) **	6.39 (3.96, 10.5) ***	1.80 (1.58, 2.05) ***
MR-proADM	Median, nmol/l	0.37	0.50	0.66	0.86	0.46
	Cases/controls (%)	32/3460 (0.9%)	98/4299 (2.3%)	58/971 (6.0%)	108/301 (35.9%)	296/9031 (3.3%)
	Model 1	Ref	1.86 (1.25, 2.84) **	4.52 (2.83, 7.35) ***	28.8 (18.1, 46.8) ***	2.93 (2.59, 3.32) ***
	Model 2	Ref	2.02 (1.35, 3.12) ***	5.04 (3.08, 8.36) ***	34.6 (20.8, 58.6) ***	3.04 (2.65, 3.48) ***

^a^Logistic regression was used to estimate OR and 95% CI of prevalent CKD for G2-4 compared with G1 of markers, as well as for per 1 SD increase in log-transformed markers. Only results for CKDcr-cys are presented in this table, while results for all three CKD assessments are presented in Table S7.

^b^Categories of NT-proBNP: G1: < 48; G2: 48–125; G3: 125–300; G4: ≥ 300 pg/ml. Categories of MR-proANP: G1: < 40; G2: 40–80; G3: 80–120; G4: ≥ 120 pmol/l. Categories of MR-proADM: G1: < 0.425; G2: 0.425–0.609; G3: 0.609–0.766; G4: ≥ 0.766 nmol/l.

Model 1: adjusted for age, sex, and study cohort;

Model 2: model 1 plus body mass index, smoking status, alcohol consumption, systolic blood pressure, use of antihypertensive medication, high-density lipoprotein cholesterol, log-transformed triglycerides, diabetes, and cardiovascular diseases.

CI, confidence interval; CKD, chronic kidney disease; CKDcr-cys, creatinine and cystatin C-based CKD; G, group; MR-proADM, mid-regional pro-adrenomedullin; MR-proANP, mid-regional pro-atrial natriuretic peptide; NT-proBNP, N-terminal pro-B-type natriuretic peptide; OR, odds ratio; Ref, reference; SD, standard deviation.

* p < 0.05, ** p < 0.01, *** p < 0.001.

### Longitudinal associations

**Fig. 2:**
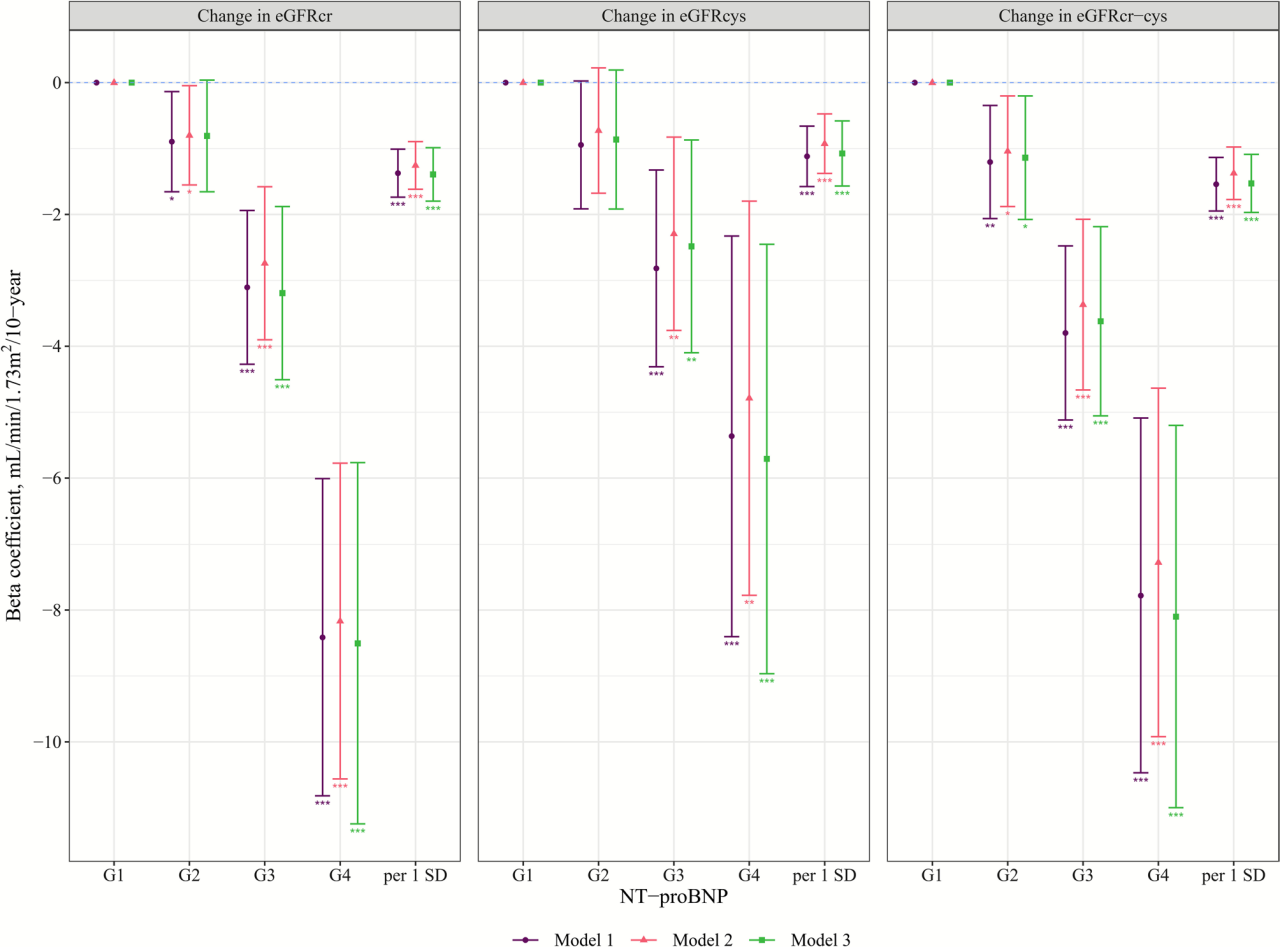
Longitudinal associations of NT-proBNP with 10-year change in kidney function. Linear mixed-effects model was used to estimate beta coefficients and 95% CI of the change in eGFR for G2-4 compared with G1 of NT-proBNP, as well as for per 1 SD increase in log-transformed NT-proBNP. The follow-up duration was used as a timescale and divided by 10 to give a 10-year change. Detailed information on adjusted models is described in Table 4. A maximum of 4205 participants with 10,208 observations were included in these analyses. Categories of NT-proBNP: G1: < 48; G2: 48–125; G3: 125–300; G4: ≥ 300 pg/ml. CI, confidence interval; G, group; eGFR, estimated glomerular filtration rate; eGFRcr, creatinine-based eGFR; eGFRcys, cystatin C-based eGFR; eGFRcr-cys, creatinine and cystatin C-based eGFR; NT-proBNP, N-terminal pro-B-type natriuretic peptide; SD, standard deviation; ^*^ p < 0.05, ^**^ p < 0.01, ^***^ p < 0.001.

**Fig. 3:**
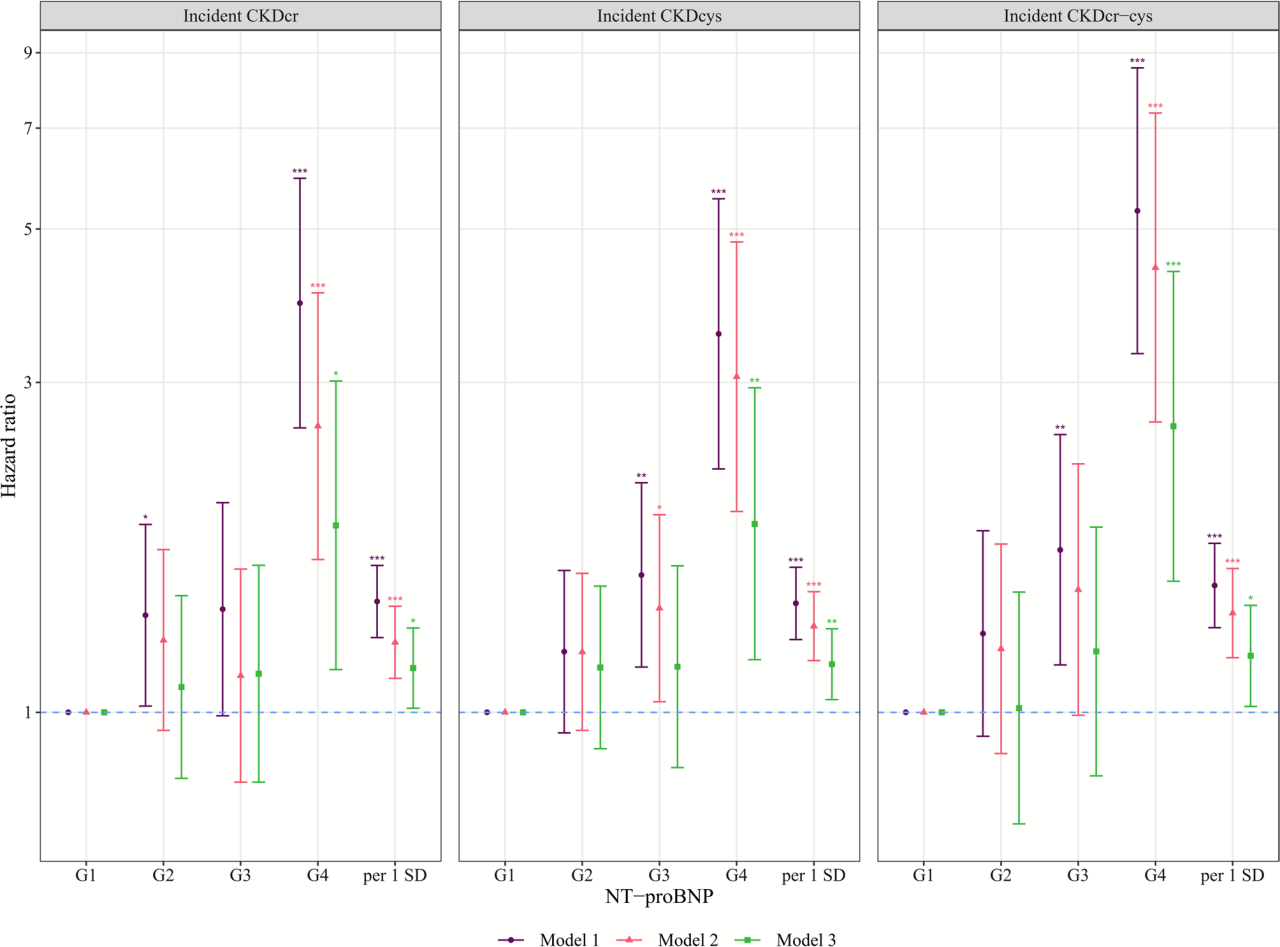
Longitudinal associations of NT-proBNP with incident CKD. Interval-censored Cox regression was used to estimate HR and 95% CI (1000 bootstrap samples for 95% CI estimation) of incident CKD for G2-4 compared with G1 of NT-proBNP, as well as for per 1 SD increase in log-transformed NT-proBNP. A total of 4167 participants free of CKDcr, 2557 free of CKDcys, and 2621 free of CKDcr-cys at baseline were included in these analyses. Detailed results are presented in Table S9. Categories of NT-proBNP: G1: < 48; G2: 48–125; G3: 125–300; G4: ≥ 300 pg/ml. CI, confidence interval; CKD, chronic kidney disease; CKDcr, creatinine-based CKD; CKDcys, cystatin C-based CKD; CKDcr-cys, creatinine and cystatin C-based CKD; G, group; HR, hazard ratio; NT-proBNP, N-terminal pro-B-type natriuretic peptide; SD, standard deviation; ^*^ p < 0.05, ^**^ p < 0.01, ^***^ p < 0.001.

Participants with higher baseline NT-proBNP levels had faster eGFR declines during follow-up (Table 4 & Fig. 2). In model 2, the β (95% CIs) of a 10-year decline in eGFRcr-cys per 1 SD increase in log-transformed NT-proBNP were −1.37 (−1.77, −0.98) ml/min/1.73m^2^/10-year, and −1.04 (−1.88, −0.20), −3.37 (−4.66, −2.07), and −7.28 (−9.92, −4.64) for G2-4 compared with G1. Regarding incident CKD, we observed 179 incident CKDcr-cys cases/30,856 person-years during follow-up. Baseline NT-proBNP levels were positively associated with incident CKD across all three incident CKD assessments (Table S9 & Fig. 3). In model 2, hazard ratio (95% CIs) was 1.39 (1.20, 1.62) per 1 SD increase in log-transformed NT-proBNP for incident CKDcr-cys. For categories of NT-proBNP, G4 shows significantly higher risks of incident CKD compared with G1 of NT-proBNP, with hazard ratio of 4.40 (2.63, 7.36) for incident CKDcr-cys. In model 3, further adjusting for baseline eGFR in longitudinal analyses, all significant associations of log-transformed NT-proBNP and G4 compared with G1 remained significant (Table 4 & Table S9).

**Table 4:** Longitudinal associations of NT-proBNP with 10-year change in kidney function^a^.

eGFR	Items	Categories of NT-proBNP^b^				per 1 SD increase in log-transformed
		**G1**	**G2**	**G3**	**G4**	
eGFRcr	Median, pg/ml	25.7	72.2	166.2	477.6	46.0
	participants; observations	2178; 5330	1494; 3622	445; 1055	88; 201	4205; 10,208
	Model 1	Ref	−0.90 (−1.66, −0.14) *	−3.11 (−4.27, −1.94) ***	−8.41 (−10.8, −6.01) ***	−1.37 (−1.74, −1.01) ***
	Model 2	Ref	−0.80 (−1.55, −0.05) *	−2.74 (−3.90, −1.58) ***	−8.17 (−10.6, −5.77) ***	−1.26 (−1.62, −0.89) ***
	Model 3	Ref	−0.81 (−1.66, 0.04)	−3.19 (−4.51, −1.88) ***	−8.51 (−11.2, −5.77) ***	−1.39 (−1.80, −0.99) ***
eGFRcys	Median, pg/ml	25.3	72.7	161.8	518.0	47.2
	participants; observations	1345; 3643	948; 2520	295; 747	63; 151	2651; 7061
	Model 1	Ref	−0.95 (−1.92, 0.02)	−2.82 (−4.31, −1.33) ***	−5.36 (−8.40, −2.33) ***	−1.12 (−1.58, −0.66) ***
	Model 2	Ref	−0.73 (−1.68, 0.22)	−2.29 (−3.76, −0.83) **	−4.79 (−7.78, −1.80) **	−0.93 (−1.38, −0.48) ***
	Model 3	Ref	−0.86 (−1.92, 0.19)	−2.48 (−4.10, −0.87) **	−5.71 (−8.97, −2.45) ***	−1.08 (−1.57, −0.58) ***
eGFRcr-cys	Median, pg/ml	25.3	72.7	161.8	518.0	47.2
	participants; observations	1345; 3643	948; 2520	295; 747	63; 151	2651; 7061
	Model 1	Ref	−1.21 (−2.06, −0.35) **	−3.80 (−5.12, −2.48) ***	−7.78 (−10.5, −5.09) ***	−1.54 (−1.95, −1.14) ***
	Model 2	Ref	−1.04 (−1.88, −0.20) *	−3.37 (−4.66, −2.07) ***	−7.28 (−9.92, −4.64) ***	−1.37 (−1.77, −0.98) ***
	Model 3	Ref	−1.14 (−2.08, −0.20) *	−3.62 (−5.06, −2.18) ***	−8.10 (−11.0, −5.20) ***	−1.53 (−1.97, −1.09) ***

^a^Linear mixed-effects models were used to investigate the associations of NT-proBNP with change in eGFR, using “lme4” package. The follow-up duration was used as the timescale and divided by 10 to give a 10-year change. The fixed effects included standardized log-transformed NT-proBNP, follow-up duration, and their interaction terms, while random effects included random intercept and random slope (i.e., individual differences in eGFR change). The β coefficient of the interaction term is the impact of NT-proBNP on the 10-year change in eGFR. The same models were applied for grouped NT-proBNP (G1-4), with G1 serving as the reference group.

^b^Categories of NT-proBNP: G1: < 48; G2: 48–125; G3: 125–300; G4: ≥ 300 pg/ml.

Model 1: adjusted for age and sex;

Model 2: model 1 plus body mass index, smoking status, alcohol consumption, systolic blood pressure, use of antihypertensive medication, high-density lipoprotein cholesterol, log-transformed triglycerides, diabetes, and cardiovascular diseases;

Model 3: model 2 plus baseline eGFR.

CI, confidence interval; G, group; eGFR, estimated glomerular filtration rate; eGFRcr, creatinine-based eGFR; eGFRcys, cystatin C-based eGFR; eGFRcr-cys, creatinine and cystatin C-based eGFR; NT-proBNP, N-terminal pro-B-type natriuretic peptide; Ref, reference; SD, standard deviation.

* p < 0.05, ** p < 0.01, *** p < 0.001.

### Stratified analysis by CVD/diabetes

**Fig. 4:**
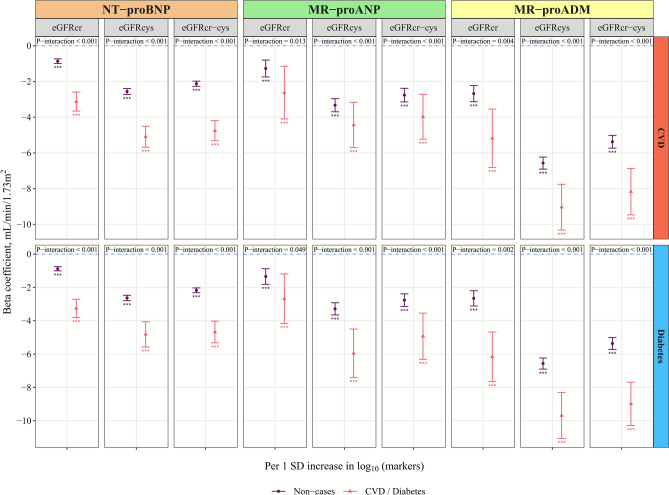
Cross-sectional associations of 3 myocardial stress markers with kidney function stratified by CVD and diabetes. Interaction terms of standardized log-transformed markers with CVD or diabetes were included in linear regression, applying model 2 described in Table 2, to test the significance of interaction. Data from 61,830 participants for NT-proBNP, 9499 for MR-proANP, and 9327 for MR-proADM were included in these analyses. Please refer to Table S10-12 for detailed results. CVD, cardiovascular disease; eGFR, estimated glomerular filtration rate; eGFRcr, creatinine-based eGFR; eGFRcys, cystatin C-based eGFR; eGFRcr-cys, creatinine and cystatin C-based eGFR; MR-proADM, mid-regional pro-adrenomedullin; MR-proANP, mid-regional pro-atrial natriuretic peptide; NT-proBNP, N-terminal pro-B-type natriuretic peptide; SD, standard deviation; ^*^ p < 0.05, ^**^ p < 0.01, ^***^ p < 0.001.

Significant interactions with both CVD and diabetes were mainly found in the cross-sectional analyses with eGFR (P-interaction < 0.05, Fig. 4). In stratified analyses, significant inverse associations of three markers with eGFR were observed in both participants with and without CVD or diabetes, while the associations were stronger among participants with diabetes or CVD (Table S10-S12). For example, the β (95% CIs) of eGFRcr-cys per 1 SD increase in log-transformed NT-proBNP were −2.13 (−2.27, −1.98) ml/min/1.73m^2^ among participants without CVD and −4.76 (−5.32, −4.20) among participants with CVD, respectively. Similar results were observed for MR-proANP and MR-proADM. For interaction analysis for prevalent CKD, only a few significant interactions were observed (Figure S5). For instance, interaction with diabetes among associations of MR-proADM with CKDcr-cys, and the associations were stronger among participants with diabetes. In longitudinal analysis, although significant interactions of NT-proBNP with CVD were observed among associations with incident CKDcr-cys (P-interaction < 0.05, Figure S6), there was no significant difference in associations among participants with and without CVD, probably due to small sample size in participants with CVD.

### Non-linear associations

A modest non-linear association of NT-proBNP with the prevalence of CKDcr-cys in cross-sectional analysis was observed (Figure S7, P-nonlinear = 0.012). The shape of the associations tended to be steeper after reaching an NT-proBNP level of around 200 pg/ml. For MR-proADM and MR-proANP, the shape of the associations also tended to be steeper for marker levels above G3, but with P-nonlinear > 0.05. Investigating NT-proBNP and incident CKD in the longitudinal analyses found no evidence of nonlinearity (Figure S8, P-nonlinear > 0.05).

## Discussion

In the present study, we investigated the associations of MR-proADM, MR-proANP, and NT-proBNP with kidney function and CKD in the general population based on pooled individual-level data from several large population-based studies. Cross-sectional analysis found that higher levels of these markers were associated with lower kidney function and a higher prevalence of CKD. Similarly, longitudinal analysis based on the MONICA/KORA study found that higher baseline NT-proBNP levels were associated with faster kidney function decline and a higher incidence of CKD. We observed significant interaction effects with CVD and diabetes mainly in the cross-sectional analyses of kidney function, with the associations being more pronounced among participants with CVD or diabetes. To the best of our knowledge, the present study has the largest sample size among cross-sectional studies in the field.

Our findings for NT-proBNP are consistent with most previous studies. A European general population-based study consisting of 8121 participants found an inverse cross-sectional association between serum NT-proBNP and kidney function^41^. Similar associations have also been observed in a cross-sectional study based on the Northern Sweden MONICA Study (N = 10,185)^42^, and two small cross-sectional studies among patients with hypertension (N = 207)^43^ or acute HF (N = 138)^44^. Several community-based cohort studies, including two Japanese^20,21^ and one American studies^22^, have also reported positive associations of serum/plasma NT-proBNP with faster kidney function decline and CKD incidence. Another cohort study reported a positive association of baseline plasma NT-proBNP with incident ESKD^23^. Various cohort studies among CKD patients have reported positive associations of circulating NT-proBNP with incident ESKD and other adverse CKD outcomes^24–30^. Collectively, circulating NT-proBNP exhibits strong associations with kidney function and kidney diseases.

Findings for MR-proANP and MR-proADM are also consistent with previous studies. A cohort study comprising 294 Japanese residents reported a positive association of plasma ANP with incident CKD^19^. Another cohort found that plasma MR-proANP and MR-proADM were positively associated with the progression of CKD among 177 CKD patients from three European countries^16^. Positive associations of MR-proADM with severe kidney outcomes such as incident ESKD have also been reported in cohort studies conducted among patients with diabetes^17,18^. Two clinical trials have reported that starting human ANP infusion at the beginning of heart bypass surgery improves postoperative kidney function in patients, regardless of their CKD status^45,46^. In another clinical trial, HF patients treated with the angiotensin-neprilysin inhibition LCZ696, a drug that suppresses the RAAS and increases natriuretic peptides levels, had a significantly lower mortality rate and a non-significantly lower kidney dysfunction incidence, compared to those treated with enalaprilin^47^. The above findings indicate the potential of MR-proANP and MR-proADM as biomarkers of kidney function decline.

In the present study, in extension to previous work, significant interactions of MR-proADM, MR-proANP, and NT-proBNP with CVD and diabetes on the cross-sectional associations with kidney function were observed, with the associations being more pronounced among participants with CVD and diabetes. This could be partially explained by the fact that both CVD and diabetes are important risk factors for the development of CKD, and thus, these individuals may be more susceptible to the impact of these markers on kidney function^38,48^. Results of non-linear associations could also support CVD-stratified results. Although no convincing non-linear associations were confirmed, the shapes of associations tended to become steeper with increasing marker levels. Since higher levels of these markers are strongly associated with CVD, the observed steeper associations with higher marker levels were similar to CVD-stratified results. Our findings suggest the importance of monitoring these markers, particularly in individuals with CVD or diabetes.

The precise mechanisms remain elusive. The elevation of these markers, as well as their active forms ADM, ANP, and BNP in the circulation, can be attributed to conditions such as ventricular/atrial wall stretch and volume overload. ADM, ANP, and BNP counteract these conditions through multiple effects, including vasodilatory, natriuretic, and diuretic effects, partially by inhibiting the actions of RAAS^7,8,12^. Chronic and excessive presence of these conditions can result in impaired cardiac function, such as cardiac output decrease, thereby contributing to an overactivation of RAAS, a key player in CKD development^11^. On the other hand, kidney function decline can lead to an accumulation of these markers in the circulation^12–15^. Nonetheless, compelling evidence has suggested that cardiac pathology remains the primary determinant for elevation of these markers^8,49^. Given the intricate interplay between the heart and kidneys, these markers may serve as valuable biomarkers for disorders involving both organs, such as the cardiorenal syndrome.

Key strengths of our study include the largest sample size derived from the general population in the cross-sectional analysis, and the estimation of kidney function using creatinine and/or cystatin C. Several limitations should also be acknowledged. First, in longitudinal analysis, data were only available in a restricted dataset and only baseline NT-proBNP was considered, and thus, we were unable to explore the impact of dynamic changes in NT-proBNP over time. Second, the counterintuitively inverse associations between obesity and levels of natriuretic peptides (e.g., MR-proANP and NT-proBNP), as reported in previous studies^50^, may have had impacts on our observed associations, despite our adjustment for body mass index. Third, the definition of CKD cases using a single creatinine and/or cystatin C measurement, and different assays across surveys, as well as the lack of follow-up confirmatory tests after a certain period such as three months, could potentially result in misclassification. However, sensitivity analysis of redefined CKD showed robust results. In addition, this study was conducted in a general population, and consequently, our data are not well-suited to establish clinically applicable cut-off values for these markers in relation to CKD. Fourth, analyses of MR-proADM and MR-proANP were only based on cross-sectional data, but our study contributes significantly to the existing literature due to its large general population-based sample size. Finally, data on albuminuria, hemoglobin A1c, specific classes of antihypertensive or antidiabetic medications, and inflammatory markers were not uniformly available across cohorts, and thus, residual confounding by these factors can not be excluded. In addition, unmeasured or uncontrolled confounders (e.g., medications that affect both the levels of these makers and kidney function) may have had impacts on the observed associations, but our sensitivity analyses and E-values suggested that our results were robust to potential unmeasured or uncontrolled confounders (Table S13-15).

In conclusion, our study found cross-sectional associations of higher levels of MR-proADM, MR-proANP, and NT-proBNP with lower kidney function and a higher prevalence of CKD, with the associations being more pronounced among individuals with CVD and diabetes. Moreover, higher NT-proBNP levels were also associated with faster kidney function decline and a higher incidence of CKD. Our findings indicate the potential utility of these myocardial stress markers as valuable biomarkers of kidney health, particularly in the context of comorbidities such as CVD and diabetes. Further research is warranted to include additional unmeasured or uncontrolled confounders and understand the underlying mechanisms driving these associations. Moreover, future studies with standardized CKD measurements in clinical settings are needed to establish clinically applicable cut-off values for these myocardial stress markers.

## Supplementary Information

Supplementary Information.

## Data Availability

The MORGAM/BiomarCaRE data are not available in a public repository. Access to the data is restricted by the ethical approvals and the legislation of the European Union and the countries of each study. Approval by the Principal Investigator of each cohort study and the MORGAM/BiomarCaRE Steering Group will be required for release of the data. The MORGAM Manual at https://www.thl.fi/publications/morgam/manual/contents.htm gives more information on access to the data. The informed consent given by MONICA/KORA study participants does not cover data posting in public databases. Cooperation partners can obtain permission to use MONICA/KORA data under the terms of a project agreement (https://helmholtz-muenchen.managed-otrs.com/external).

## Abbreviations

BiomarCaRE: Biomarkers for Cardiovascular Risk Assessment in Europe
CI: Confidence interval
CKD: Chronic kidney disease
CKD-EPI: Chronic kidney disease epidemiology collaboration
cr: Creatinine-based
cr-cys: Combined creatinine and cystatin C-based
CVD: Cardiovascular disease
cys: Cystatin C-based
eGFR: Estimated glomerular filtration rate
ESKD: End-stage kidney disease
G: Group
HF: Heart failure
KORA: Cooperative Health Research in the Region of Augsburg
MONICA: Monitoring of Trends and Determinants in Cardiovascular Diseases
MORGAM: Monitoring of Trends and Determinants in Cardiovascular Diseases Risk, Genetics, Archiving and Monograph
MR-proADM: Mid-regional pro-adrenomedullin
MR-proANP: Mid-regional pro-atrial natriuretic peptide
NT-proBNP: N-terminal pro-B-type natriuretic peptide
OR: Odds ratio
RAAS: Renin–angiotensin–aldosterone system
SD: Standard deviation
