# Supplementary material for: Longitudinal and cross-sectional associations of myocardial stress markers with kidney function and chronic kidney disease in the BiomarCaRE project
Source: Sci Rep. 2026 Feb 23;16:7488. doi: 10.1038/s41598-026-37377-2 (PMC12929796; doi:10.1038/s41598-026-37377-2)
Supplement: Supplementary file 1 — Supplementary Information. [file 41598_2026_37377_MOESM1_ESM.pdf]

Supplementary materials

Longitudinal and cross-sectional associations of myocardial stress markers with kidney function and chronic kidney disease in the BiomarCaRE project

Jie-sheng Lin, Tanja Zeller, Wolfgang Koenig, Pekka Jousilahti, Frank Kee, Licia Iacoviello, Hugh Tunstall-Pedoe, Stefan Söderberg, Giancarlo Cesana, Luigi Palmieri, Veikko Salomaa, Julia de Man Lapidoth, Roberto De Ponti, Chiara Donfrancesco, Thiess Lorenz, Kari Kuulasmaa, Stefan Blankenberg, Annette Peters, Barbara Thorand

Table of Contents

Supplementary texts 3

Text S1. Details of laboratory measurements and kidney function assessment 3

Text S2. Inverse probability weighting 5

Supplementary tables 6

Table S1. Overview and description of contributing studies/cohorts in BiomarCaRE 6

Table S2. Intra-assay and inter-assay coefficients of variation for laboratory measurements in included study cohorts 8

Table S3. Baseline characteristics of participants included in the cross-sectional analysis across study cohorts 9

Table S4. Characteristics of participants with data on MR-proADM and MR-proANP 10

Table S5. Baseline characteristics of participants from the MONICA/KORA study included in the longitudinal analysis 11

Table S6. Cross-sectional associations of 3 myocardial stress markers with kidney function 12

Table S7. Cross-sectional associations of 3 myocardial stress markers with CKD 14

Table S8. Sensitivity analysis of cross-sectional associations of 3 myocardial stress markers with CKD 16

Table S9. Longitudinal associations of NT-proBNP with incident CKD 17

Table S10. Cross-sectional associations of NT-proBNP with kidney function stratified by CVD and diabetes 18

Table S11. Cross-sectional associations of MR-proANP with kidney function stratified by CVD and diabetes 19

Table S12. Cross-sectional associations of MR-proADM with kidney function stratified by CVD and diabetes 20

Table S13. Sensitivity analysis of cross-sectional associations of 3 myocardial stress markers with eGFRcr-cys and CKDcr-cys 21

Table S14. Sensitivity analysis of longitudinal associations of NT-proBNP with change in eGFRcr-cys and incident CKDcr-cys 22

Table S15. E-values to assess the robustness of observed associations to potential unmeasured or uncontrolled confounders 23

Supplementary figures 24

Figure S1. Flowchart of study participants from the MONICA/KORA study included in the longitudinal analysis 24

Figure S2. Cross-sectional associations of 3 myocardial stress markers with kidney function 25

Figure S3. Cross-sectional associations of 3 myocardial stress markers with CKD 26

Figure S4. Sensitivity analysis of cross-sectional associations of 3 myocardial stress markers with CKD 27

Figure S5. Cross-sectional associations of 3 myocardial stress markers with CKD stratified by CVD and diabetes 28

Figure S6. Longitudinal associations of NT-proBNP with 10-year change in kidney function and incident CKD stratified by CVD and diabetes 29

Figure S7. Shapes of the associations between 3 myocardial stress markers and prevalent CKDcr-cys 30

Figure S8. Shapes of the associations between NT-proBNP and incident CKD 31

References 32

Supplementary texts

Text S1. Details of laboratory measurements and kidney function assessment

In BiomarCaRE/MORGAM, plasma/serum samples were used to measure the following markers and indices: Plasma MR-proADM and MR-proANP (only available in study FINRISK and PRIME/Belfast) were measured on the BRAHMS KRYPTOR automated system with immunoluminometric assay (BRAHMS/Thermo Fisher Scientific, Hennigsdorf, Berlin, Germany). Serum NT-proBNP was measured on the ELECSYS 2010 or the Cobas e411 using an electrochemiluminescence immunoassay (ECLIA, Roche Diagnostics, Mannheim, Germany). Plasma/serum creatinine was measured by the kinetic alkaline picrate Jaffe method on the Abbott Architect c8000 with the isotope dilution mass spectrometry (IDMS) traceable (NIST SRM 967) Abbott Architect Assay CREATININE. Serum cystatin C was measured on the Abbott Architect c8000 with the Latex immunoassay. High-density lipoprotein cholesterol, low-density lipoprotein cholesterol, and triglycerides were measured locally by routine methods.

In the baseline of MONICA/KORA study (S3 & S4), part of creatinine was measured by the enzymatic method (CREA plus, Boehringer, Mannheim, Germany). In follow-up surveys of MONICA/KORA study, creatinine was assessed in serum using a modified kinetic rate Jaffe method at MONICA/KORA F3 & F4 and the first part of FF4 (CREA Flex, Dade Behring / Siemens Healthcare Diagnostics Products GmbH), and a Jaffe method for the second part of FF4 (Cobas 8000 instrument, Roche Diagnostics, Mannheim, Germany). Serum creatinine at MONICA/KORA F3 &F4 and FF4 (part 1) were Isotope-Dilution Mass Spectrometry standardized. Serum cystatin C was measured using N Latex Cystatin C assay (Siemens Healthcare Diagnostics Products GmbH) using particle-enhanced immunonephelometry at MONICA/KORA F3/F4/FF4. Cystatin C at F3/F4 was calibrated to the International Federation of Clinical Chemistry and Laboratory Medicine standard by multiplying by a factor of 1.174 as suggested by Siemens.

Kidney function was assessed by estimated glomerular filtration rate (eGFR), with creatinine-based (eGFRcr), cystatin C-based eGFR (eGFRcys), and combined creatinine and cystatin C-based eGFR (eGFRcr-cys), calculated using the following Chronic Kidney Disease Epidemiology Collaboration (CKD-EPI) equations:

The CKD-EPI Creatinine Equation 2021 [1]:

eGFRcr = $142 \times \min(\mathbf{cr}/k,1)^{\alpha} \times \max(\mathbf{cr}/k,1)^{-1.200} \times 0.9938^{Age} \times 1.012$ [if female], where **cr** is creatinine, k is 0.7 for females and 0.9 males, α is -0.241 for females and -0.302 for males, min indicates the minimum of cr/k or 1, max indicates the maximum of cr/k or 1.

The CKD-EPI Cystatin C Equation 2012 [2]:

eGFRcys = $133 \times \min(\mathbf{cys}/0.8,1)^{-0.499} \times \max(\mathbf{cys}/0.8,1)^{-1.328} \times 0.9962^{Age} \times 0.932$ [if female], where **cys** is cystatin C, min indicates the minimum of cys/0.8 or 1, max indicates the maximum of cys/0.8 or 1.

The CKD-EPI Creatinine-Cystatin C Equation 2021 [1]:

eGFRcr-cys = $135 \times \min(\mathbf{cr}/k,1)^{\alpha} \times \max(\mathbf{cr}/k,1)^{-0.544} \times \min(\mathbf{cys}/0.8,1)^{-0.323} \times \max(\mathbf{cys}/0.8,1)^{-0.778} \times 0.9961^{Age} \times 0.963$ [if female], where **cr** is creatinine, **cys** is cystatin C, k is 0.7 for females and 0.9 males, α is -0.219 for females and -0.144 for males, min indicates the minimum of cr/k or 1 and the minimum of cys/0.8 or 1, max indicates the maximum of cr/k or 1 and the maximum of cys/0.8 or 1.

Text S2. Inverse probability weighting

To partially address bias caused by loss to follow-up in the longitudinal analysis (due to death or other reasons, e.g., refusal or inability to contact, Figure S1), the inverse probability weighting-weights were applied in all longitudinal analyses in the present study. Each participant's probability of loss to follow-up (P1) was estimated by logistic regression model with loss to follow-up (yes/no) as outcomes, including baseline age, sex, body mass index, smoking status, alcohol consumption, systolic blood pressure, use of antihypertensive medication, log-transformed triglycerides, high-density lipoprotein-cholesterol, diabetes, cardiovascular diseases, creatinine-based estimated glomerular filtration rate, and standardized log-transformed NT-proBNP as predictors. Inverse probability weighting-weight was calculated as 1/(1-P1). Then the weight was applied in longitudinal analyses of associations of NT-proBNP with the change in kidney function and incident chronic kidney disease.

Supplementary tables

Table S1. Overview and description of contributing studies/cohorts in BiomarCaRE

Study cohort [Reference]	Country	Study/cohort full name and short description
FINRISK [3]	Finland	The FINRISK study is a series of population-based cardiovascular risk factor surveys carried out every five years in five (or six in 2002) districts of Finland, including North Karelia, Northern Savo (former Kuopio), Southwestern Finland, Oulu Province, Lapland Province (in 2002 only) and the region of Helsinki and Vantaa. A stratified random sample was drawn for each survey from the national population register; the age range was 25-74 years. All individuals enrolled in the study received a physical examination, a self-administered questionnaire, and a blood sample was drawn. For the current analysis, we used only the FINRISK 1997 survey. In 1997, altogether 11500 individuals were invited and 8444 (73%) participated in the clinical examination. The Coordinating Ethics Committee of the Helsinki and Uusimaa Hospital District approved the study, which followed the declaration of Helsinki. All subjects gave written informed consent. (Ethical approval: Ethics Committee at National Public Health Institute of Finland, 38/96). https://www.thl.fi/publications/morgam/cohorts/full/finland/fin-fina.htm
Cooperative Health Research in the Region of Augsburg (MONICA/KORA) [4, 5]	Germany	The WHO Multinational Monitoring of Trends and Determinants in Cardiovascular Diseases (MONICA)/Cooperative Health Research in the Region of Augsburg (KORA) cohorts comprise all respondents from representative sample surveys from the city of Augsburg and the less urban Landkreis Augsburg and Landkreis Aichach-Friedberg regions in Bavaria, Southern Germany. List of municipalities and population registers were used as sampling frames for the first and the second stage of two-stage sampling, respectively. The second stage of sampling was stratified by sex and ten-year age group. The Survey 3 (S3) baseline examination (1994-1995) was carried out as part of the WHO MONICA project and consisted of 4856 men and women aged 25-74 years with a response rate of 75%, and 3006 participants were followed up after 10 years (2004-2005, F3). The Survey 4 (S4) baseline examination was carried out in 1999-2001 and includes 4261 participants (response rate: 66%), and 3080 participants were followed up after 7 years (2006-2008, F4) and 2279 participants after 14 years (2013-2014, FF4). The BiomarCaRE project includes 4692 and 4221 participants from S3 and S4, respectively. (Ethical approval: Ethik-Kommission Bayerische Landesärztekammer, Nr.05004, Nr. 99186, and Nr. 06068). https://www.thl.fi/publications/morgam/cohorts/full/germany/ger-auga.htm
Moli-sani Study [6]	Italy	The cohort of the Moli-sani Study was recruited in the Molise region from city hall registries by multistage sampling. First, townships were sampled in major areas by cluster sampling; then, within each township, participants aged 35 years or over were selected by simple random sampling. Exclusion criteria were pregnancy at the time of recruitment, lack of understanding, current multiple trauma or coma, or refusal to sign the informed consent. A total of 24325 men (47%) and women (53%) over the age of 35 were examined at baseline from 2005 to 2010. The participation rate was 70%. (Ethical approval: Comitato Etico Università Cattolica del Sacro Cuore—Roma, Prot. pdc. P. 99 (A. 931/03-138-04)/C.E./2004). https://www.thl.fi/publications/morgam/cohorts/full/italy/ita-mola.htm
MONICA Brianza Study [7]	Italy	The MONICA-Brianza Cohort Study is a prospective study of three cohorts of 25-64 years old residents in Brianza, a highly-industrialized area located between Milan and the Swiss border, Northern Italy. Gender- and ten-year age-stratified samples were randomly drawn in 1986, 1990, and 1993, and cardiovascular risk factors were investigated at baseline following the procedures of the WHO MONICA Project. The overall participation rate was 69%. For all subjects' whole blood and serum samples were stored in a biobank. (Ethical approval: Comitato Etico Azienda Ospedaliera San Gerardo—Monza, 192/2005). https://www.thl.fi/publications/morgam/cohorts/full/italy/ita-bria.htm

Study cohort [Reference]	Country	Study/cohort full name and short description
Northern Sweden MONICA Study [8]	Sweden	The Northern Sweden MONICA study covered the two northernmost counties of Sweden, i.e., Norrbotten and Västerbotten with altogether 510,000 inhabitants. Population surveys performed in 1986, 1990, 1994, 1999, 2004, and 2009, with altogether 10,517 unique participants, were included in the present study. On the first two occasions, 2,000 persons aged 25 to 64 years were randomly selected, and in the last three surveys, the upper age limit was extended to 74 years, and 2,500 individuals were invited. A stratified randomized selection procedure by age and sex (250 persons in each sex/10-year age stratum) has been used. The participation rate was 69-81%. (Ethical approval: Research ethic Committee of Umea University, 2012-280-32M). https://www.thl.fi/publications/morgam/cohorts/full/sweden/swe-nswa.htm https://www.umu.se/forskning/projekt/monica-studien/
Prospective Epidemiological Study of Myocardial Infarction (PRIME) Belfast [9]	United Kingdom	The PRIME/Belfast study examined the classic and putative cardiovascular risk factors to explain the large difference in heart disease incidence in Ireland. The study includes men aged 50-59 from Belfast, Northern Ireland (N=2745). Baseline examinations took place in 1990-1993 and targeted cohorts which had broadly similar social class structures to the background population, initially sampling from industries and various employment groups, employment groups with more than 10% of their workforce of foreign origin were excluded. (Ethical approval: Office for Research Ethics Committees Northern Ireland, 06/NIR02/107). https://www.thl.fi/publications/morgam/cohorts/full/uk/unk-bela.htm
Scottish Heart Health Extended Cohort (SHHEC) [10]	United Kingdom	This consists of two overlapping studies which share a common protocol and methods: the Scottish Heart Health Study randomly recruited men and women aged 40-59 across 22 Scottish districts in 1984-1987; Scottish MONICA similarly recruited men and women aged 25-64 in Edinburgh and North Glasgow in 1986, and in North Glasgow again in 1989, 1992 (up to 74), and in 1995 as part of the WHO MONICA Project. They are now combined as one cohort. (Ethical approval: Tayside Health Board Dundee District, DM/CL/207). https://www.thl.fi/publications/morgam/cohorts/full/uk/unk-sco.htm

Table S2. Intra-assay and inter-assay coefficients of variation for laboratory measurements in included study cohorts

Study cohort	Creatinine (N = 61830)		Cystatin C (N = 61830)		NT-proBNP (N = 61830)		MR-proADM (N = 9327)[a]		MR-proANP (N = 9499)[a]	
	Intra-assay (%)	Inter-assay (%)	Intra-assay (%)	Inter-assay (%)	Intra-assay (%)	Inter-assay (%)	Intra-assay (%)	Inter-assay (%)	Intra-assay (%)	Inter-assay (%)
FINRISK	0-0.09	2.28-4.69	3.34	1.80-3.31	2.58	1.38	2.17	2.43	3.65	2.33
MONICA/KORA	0.97-2.74	4.22-6.44	1.03-1.17	6.82-7.79	1.17	5.52-9.18				
MATISS	1.20-2.53	4.10-5.4	0.80-2.46	4.60-5.00	3.23	6.30-8.80				
Moli-sani	0.76-1.55	4.56-6.96	4.10-6.2	1.09-1.75	2.30	5.44-6.50				
MONICA_Brianza	0.97	2.36-4.65	1.04	4.04-5.76	2.64	3.28-5.20				
Northern Sweden MONICA	0.67-5.2	5.78-8.07	0.78-2.78	4.10-12.47	1.48	5.88-8.70				
PRIME/Belfast	0-0.09	2.28-4.69	3.34	1.80-3.31	2.58	1.38	2.17	2.43	3.65	2.33
SHHEC [b]	1.25	4.68	NA	NA	1.13-1.17	6.63-6.73				

[a] MR-proADM and MR-proANP are only available in study FINRISK and PRIME/Belfast.
[b] Values of intra-assay and inter-assay of cystatin C are unavailable in SHHEC.
Abbreviations: MR-proADM, mid-regional pro-adrenomedullin; MR-proANP, mid-regional pro-atrial natriuretic peptide; NT-proBNP, N-terminal pro-B-type natriuretic peptide.

Table S3. Baseline characteristics of participants included in the cross-sectional analysis across study cohorts

Characteristics	FINRISK (N = 6858)	MONICA/KORA (N = 5790)	Moli-sani (N = 22243)	MONICA_Brianza (N = 3623)	Northern Sweden MONICA (N = 10414)	PRIME/Belfast (N = 1539)	SHHEC (N = 11363)
	Mean (standard deviation) or number (%)						
Age (years)	48.8 (13.3)	48.0 (13.7)	56.2 (12.0)	46.8 (11.3)	50.0 (13.6)	54.7 (2.9)	49.6 (9.2)
Sex, N (%) female	3588 (52.3)	3071 (53.0)	11938 (53.7)	1939 (53.5)	5668 (54.4)	0 (0.0)	6005 (52.8)
Body mass index (kg/m^2)	26.7 (4.61)	27.2 (7.75)	28.1 (5.18)	26.1 (8.48)	27.4 (7.08)	26.2 (3.46)	26.0 (5.52)
Current Smoker, N (%)	1781 (26.0)	1562 (27.0)	4943 (22.2)	1147 (31.7)	2331 (22.4)	440 (28.6)	5025 (44.2)
Alcohol consumption, N (%)							
No alcohol consumption	2698 (39.3)	1789 (30.9)	6857 (30.8)	1420 (39.2)	3501 (33.6)	627 (40.7)	3373 (29.7)
>0 and <20 g/day	3335 (48.6)	2161 (37.3)	8869 (39.9)	0 (0.0)	6770 (65.0)	341 (22.2)	5525 (48.6)
≥ 20 g/day	825 (12.0)	1840 (31.8)	6517 (29.3)	2203 (60.8)	143 (1.4)	571 (37.1)	2465 (21.7)
Systolic blood pressure (mm Hg)	136.5 (25.0)	131.8 (48.3)	141.4 (24.6)	134.9 (65.9)	130.2 (32.6)	134.5 (30.5)	132.0 (20.4)
Use of antihypertensive medication, N (%)	983 (14.3)	743 (12.8)	6589 (29.6)	397 (11.0)	1352 (13.0)	155 (10.1)	877 (7.7)
Hypertension, N (%)	3132 (45.7)	2052 (35.4)	12613 (56.7)	1300 (35.9)	3616 (34.7)	618 (40.2)	4243 (37.3)
HDL-cholesterol (mmol/L)	1.45 (0.36)	1.49 (0.39)	1.45 (0.34)	1.42 (0.37)	1.47 (0.45)	1.18 (0.33)	1.49 (0.44)
LDL-cholesterol (mmol/L)	3.07 (0.85)	3.47 (0.99)	3.30 (0.86)	3.26 (0.98)	3.72 (1.29)	3.81 (0.87)	3.30 (1.05)
Triglycerides (mmol/L), median [IQR]	1.16 [0.87, 1.56]	1.37 [0.99, 1.90]	1.21 [0.89, 1.66]	1.10 [0.80, 1.54]	1.16 [0.82, 1.68]	1.63 [1.17, 2.36]	1.54 [1.10, 2.16]
Diabetes, N (%)	401 (5.8)	262 (4.5)	1466 (6.6)	94 (2.6)	438 (4.2)	37 (2.4)	192 (1.7)
Cardiovascular diseases, N (%)	433 (6.3)	235 (4.1)	682 (3.1)	64 (1.8)	574 (5.5)	103 (6.7)	472 (4.2)
eGFRcr (ml/min/1.73 m^2)	91.9 (19.4)	102.4 (15.2)	95.7 (14.8)	95.8 (17.6)	102.1 (16.9)	88.3 (19.2)	98.2 (16.3)
CKDcr, N (%)	322 (4.7)	76 (1.3)	583 (2.6)	165 (4.6)	217 (2.1)	122 (7.9)	348 (3.1)
eGFRcys (ml/min/1.73 m^2)	98.8 (19.5)	99.8 (19.7)	85.8 (22.3)	93.7 (20.2)	93.8 (23.8)	83.9 (15.9)	108.4 (17.2)
CKDcys, N (%)	227 (3.3)	218 (3.8)	2860 (12.9)	202 (5.6)	954 (9.2)	81 (5.3)	121 (1.1)
eGFRcr-cys (ml/min/1.73 m^2)	98.4 (18.0)	104.5 (17.0)	93.4 (18.4)	97.3 (18.5)	101.3 (21.9)	88.4 (15.6)	108.2 (16.6)
CKDcr-cys, N (%)	202 (2.9)	79 (1.4)	980 (4.4)	136 (3.8)	382 (3.7)	64 (4.2)	113 (1.0)
NT-proBNP, (pg/ml), median [IQR]	47.4 [24.8, 87.6]	49.2 [26.1, 90.4]	50.3 [26.5, 94.2]	39.4 [20.5, 71.9]	44.5 [23.4, 83.5]	34.5 [19.2, 62.3]	52.3 [28.0, 97.6]
MR-proANP, (pmol/l), median [IQR] [a]	44.2 [33.2, 61.0]	NA	NA	NA	NA	66.8 [52.5, 87.0]	NA
MR-proADM, (nmol/l), median [IQR] [a]	0.47 [0.39, 0.57]	NA	NA	NA	NA	0.45 [0.40, 0.51]	NA

[a] MR-proADM and MR-proANP are only available in study FINRISK and PRIME/Belfast.

Abbreviations: CKD, chronic kidney disease; cr, creatinine-based; cys, cystatin C-based; cr-cys, combined creatinine and cystatin C-based; eGFR, estimated glomerular filtration rate; HDL, high-density lipoprotein; IQR, interquartile range; LDL, low-density lipoprotein; MR-proADM, mid-regional pro-adrenomedullin; MR-proANP, mid-regional pro-atrial natriuretic peptide; NT-proBNP, N-terminal pro-B-type natriuretic peptide.

Table S4. Characteristics of participants with data on MR-proADM and MR-proANP

Characteristics	Total (N = 9503)	FINRISK [a] (N = 7637)	PRIME/Belfast [a] (N = 1866)	*P*-value [b]
	Mean (standard deviation) or number (%)			
Age (years)	49.4 (12.3)	48.1 (13.3)	54.7 (2.9)	<0.001
Sex, N (%) female	3811 (40.1)	3811 (49.9)	0 (0.0)	<0.001
Body mass index (kg/m^2)	26.66 (4.50)	26.74 (4.71)	26.31 (3.49)	<0.001
Current smoker, N (%)	2554 (26.9)	2028 (26.6)	526 (28.2)	0.162
Alcohol consumption, N (%)				<0.001
No alcohol consumption	3711 (39.1)	2946 (38.6)	765 (41.0)	
>0 and <20 g/day	4143 (43.6)	3736 (48.9)	407 (21.8)	
≥ 20 g/day	1649 (17.4)	955 (12.5)	694 (37.2)	
Systolic blood pressure (mm Hg)	136.0 (26.8)	136.3 (24.3)	134.9 (35.0)	0.042
Use of antihypertensive medication, N (%)	1243 (13.1)	1062 (13.9)	181 (9.7)	<0.001
Hypertension, N (%)	4193 (44.1)	3456 (45.3)	737 (39.5)	<0.001
HDL-cholesterol (mmol/L)	1.39 (0.37)	1.44 (0.36)	1.19 (0.34)	<0.001
LDL-cholesterol (mmol/L)	3.23 (0.91)	3.08 (0.86)	3.83 (0.88)	<0.001
Triglycerides (mmol/L), median [IQR]	1.24 [0.91, 1.73]	1.17 [0.88, 1.58]	1.67 [1.19, 2.40]	<0.001
Diabetes, N (%)	484 (5.1)	435 (5.7)	49 (2.6)	<0.001
Cardiovascular diseases, N (%)	574 (6.0)	457 (6.0)	117 (6.3)	0.681
eGFRcr (ml/min/1.73 m^2)	91.3 (19.6)	92.3 (19.5)	87.0 (19.2)	<0.001
eGFRcys (ml/min/1.73 m^2)	96.2 (19.8)	99.2 (19.5)	84.1 (15.9)	<0.001
eGFRcr-cys (ml/min/1.73 m^2)	96.7 (18.1)	98.8 (18.1)	87.9 (15.5)	<0.001
NT-proBNP, (pg/ml), median [IQR]	44.5 [23.7, 82.9]	47.4 [24.8, 87.6]	34.5 [19.2, 62.4]	<0.001
MR-proANP, (pmol/l), median [IQR]	46.8 [33.9, 66.4]	42.8 [32.0, 59.6]	64.8 [50.9, 84.7]	<0.001
MR-proADM, (nmol/l), median [IQR]	0.46 [0.39, 0.55]	0.47 [0.39, 0.56]	0.45 [0.39, 0.51]	<0.001

[a] MR-proADM and MR-proANP are only available in study FINRISK and PRIME/Belfast.
[b] P-value was estimated by t-Test / Mann-Whitney U test (continuous variables) or chi-squared test (categorical variables).
Abbreviations: eGFRcr, creatinine-based estimated glomerular filtration rate; eGFRcys, cystatin C-based eGFR; eGFRcr-cys, combined creatinine and cystatin C-based eGFR; IQR, interquartile range; KORA, Cooperative Health Research in the Region of Augsburg; LDL, low-density lipoprotein; MR-proADM, mid-regional pro-adrenomedullin; MR-proANP, mid-regional pro-atrial natriuretic peptide; NT-proBNP, N-terminal pro-B-type natriuretic peptide.

Table S5. Baseline characteristics of participants from the MONICA/KORA study included in the longitudinal analysis

Characteristics	Total (N = 4167)	Non-cases (N = 3931)	Incident CKDcr (N = 236)	*P*-value [a]
	Mean (standard deviation) or number (%)			
Age (years)	46.5 (12.8)	45.6 (12.4)	61.9 (8.8)	<0.001
Sex, N (%) female	2226 (53.4)	2109 (53.7)	117 (49.6)	0.250
Body mass index (kg/m^2)	26.6 (4.36)	26.4 (4.30)	28.9 (4.65)	<0.001
Current smoker, N (%)	1008 (24.2)	980 (24.9)	28 (11.9)	<0.001
Alcohol consumption, N (%)				0.081
No alcohol consumption	1177 (28.2)	1096 (27.9)	81 (34.3)	
>0 and <20 g/day	1646 (39.5)	1565 (39.8)	81 (34.3)	
≥ 20 g/day	1344 (32.3)	1270 (32.3)	74 (31.4)	
Systolic blood pressure (mm Hg)	126.7 (17.9)	126.1 (17.5)	137.9 (20.8)	<0.001
Use of antihypertensive medication, N (%)	527 (12.6)	412 (10.5)	115 (48.7)	<0.001
Hypertension, N (%)	1315 (31.6)	1159 (29.5)	156 (66.1)	<0.001
HDL-cholesterol (mmol/L)	1.48 (0.44)	1.49 (0.44)	1.43 (0.43)	0.047
LDL-cholesterol (mmol/L)	3.50 (1.06)	3.49 (1.06)	3.78 (1.06)	<0.001
Triglycerides (mmol/L), median [IQR]	1.37 [0.99, 1.92]	1.36 [0.98, 1.92]	1.54 [1.20, 2.09]	<0.001
Diabetes, N (%)	133 (3.2)	99 (2.5)	34 (14.4)	<0.001
Cardiovascular diseases, N (%)	268 (6.4)	219 (5.6)	49 (20.8)	<0.001
eGFRcr (ml/min/1.73 m^2)	102.0 (15.0)	103.1 (14.3)	82.6 (13.2)	<0.001
eGFRcys (ml/min/1.73 m^2)	97.5 (18.1)	98.9 (17.2)	73.9 (16.1)	<0.001
eGFRcr-cys (ml/min/1.73 m^2)	102.7 (15.4)	104.0 (14.5)	80.5 (14.0)	<0.001
NT-proBNP, (pg/ml), median [IQR]	45.9 [25.1, 81.8]	44.5 [24.5, 78.2]	90.6 [47.9, 160.1]	<0.001

[a] P-value was estimated by t-Test / Mann-Whitney U test (continuous variables) or chi-squared test (categorical variables).

Abbreviations: CKDcr, creatinine-based chronic kidney disease; eGFRcr, creatinine-based estimated glomerular filtration rate; eGFRcys, cystatin C-based eGFR; eGFRcr-cys, combined creatinine and cystatin C-based eGFR; IQR, interquartile range; KORA, Cooperative Health Research in the Region of Augsburg; LDL, low-density lipoprotein; MONICA, Monitoring of Trends and Determinants in Cardiovascular Diseases; NT-proBNP, N-terminal pro-B-type natriuretic peptide.

Table S6. Cross-sectional associations of 3 myocardial stress markers with kidney function [a]

Markers	eGFR	Items	Categories of markers [b]				per 1 SD increase in log-transformed
			G1	G2	G3	G4	
**NT-proBNP**							
		Median, pg/ml	25.4	72.5	169.4	501.2	48.0
		N	30939	21284	7109	2498	61830
	**eGFRcr**						
		Model 1	Ref.	-0.06 (-0.31, 0.19)	-1.28 (-1.65, -0.90) ***	-7.24 (-7.82, -6.65) ***	-1.07 (-1.19, -0.95) ***
		Model 2	Ref.	-0.10 (-0.35, 0.14)	-1.12 (-1.49, -0.74) ***	-6.92 (-7.51, -6.34) ***	-1.06 (-1.18, -0.93) ***
	**eGFRcys**						
		Model 1	Ref.	-1.77 (-2.09, -1.45) ***	-5.34 (-5.82, -4.86) ***	-12.5 (-13.2, -11.7) ***	-2.73 (-2.89, -2.57) ***
		Model 2	Ref.	-2.03 (-2.35, -1.72) ***	-5.26 (-5.73, -4.79) ***	-11.9 (-12.6, -11.1) ***	-2.78 (-2.94, -2.63) ***
	**eGFRcr-cys**						
		Model 1	Ref.	-1.22 (-1.51, -0.94) ***	-4.23 (-4.66, -3.80) ***	-11.7 (-12.4, -11.0) ***	-2.32 (-2.47, -2.18) ***
		Model 2	Ref.	-1.41 (-1.69, -1.14) ***	-4.10 (-4.52, -3.68) ***	-11.2 (-11.8, -10.5) ***	-2.35 (-2.49, -2.21) ***
**MR-proANP**							
		Median, pmol/l	30.8	53.7	92.8	147.2	46.8
		N	3540	4493	1048	418	9499
	**eGFRcr**						
		Model 1	Ref.	-1.88 (-2.75, -1.01) ***	-2.90 (-4.26, -1.53) ***	-7.13 (-9.04, -5.22) ***	-1.53 (-1.97, -1.09) ***
		Model 2	Ref.	-1.95 (-2.82, -1.08) ***	-2.78 (-4.16, -1.40) ***	-6.53 (-8.48, -4.57) ***	-1.45 (-1.90, -1.00) ***
	**eGFRcys**						
		Model 1	Ref.	-1.84 (-2.56, -1.13) ***	-5.94 (-7.06, -4.81) ***	-14.0 (-15.6, -12.4) ***	-3.25 (-3.61, -2.89) ***
		Model 2	Ref.	-2.65 (-3.33, -1.97) ***	-6.79 (-7.88, -5.71) ***	-13.7 (-15.2, -12.1) ***	-3.49 (-3.85, -3.14) ***
	**eGFRcr-cys**						
		Model 1	Ref.	-2.16 (-2.88, -1.45) ***	-5.30 (-6.43, -4.17) ***	-12.4 (-13.9, -10.8) ***	-2.83 (-3.20, -2.47) ***
		Model 2	Ref.	-2.68 (-3.39, -1.98) ***	-5.74 (-6.86, -4.62) ***	-11.8 (-13.4, -10.2) ***	-2.93 (-3.30, -2.57) ***

Markers	eGFR	Items	Categories of markers [b]				per 1 SD increase in log-transformed
			G1	G2	G3	G4	
**MR-proADM**							
		Median, nmol/l	0.37	0.50	0.66	0.86	0.46
		N	3492	4397	1029	409	9327
	**eGFRcr**						
		Model 1	Ref.	-2.61 (-3.43, -1.79) ***	-4.68 (-6.01, -3.35) ***	-10.9 (-12.8, -9.00) ***	-2.72 (-3.12, -2.31) ***
		Model 2	Ref.	-2.85 (-3.69, -2.01) ***	-4.97 (-6.34, -3.60) ***	-11.2 (-13.2, -9.23) ***	-2.88 (-3.32, -2.45) ***
	**eGFRcys**						
		Model 1	Ref.	-6.98 (-7.61, -6.35) ***	-16.4 (-17.4, -15.4) ***	-27.5 (-29.0, -26.1) ***	-7.35 (-7.66, -7.04) ***
		Model 2	Ref.	-6.22 (-6.85, -5.60) ***	-14.8 (-15.8, -13.7) ***	-25.2 (-26.7, -23.7) ***	-6.77 (-7.10, -6.45) ***
	**eGFRcr-cys**						
		Model 1	Ref.	-5.55 (-6.20, -4.89) ***	-12.4 (-13.5, -11.4) ***	-22.2 (-23.7, -20.7) ***	-5.84 (-6.17, -5.52) ***
		Model 2	Ref.	-5.24 (-5.90, -4.58) ***	-11.7 (-12.7, -10.6) ***	-21.0 (-22.6, -19.5) ***	-5.60 (-5.94, -5.26) ***

[a] Linear regression was used to estimate beta coefficients and 95% CI of eGFR for G2-4 compared with G1 of makers, as well as for per 1 SD increase in log-transformed markers.
[b] Categories of NT-proBNP: G1: < 48; G2: 48-125; G3: 125-300; G4: ≥ 300 pg/ml. Categories of MR-proANP: G1: < 40; G2: 40-80; G3: 80-120; G4: ≥ 120 pmol/l. Categories of MR-proADM: G1: < 0.425; G2: 0.425-0.609; G3: 0.609-0.766; G4: ≥ 0.766 nmol/l.
Model 1: adjusted for age, sex, and study cohort;
Model 2: model 1 plus body mass index, smoking status, alcohol consumption, systolic blood pressure, use of antihypertensive medication, high-density lipoprotein cholesterol, log-transformed triglycerides, diabetes, and cardiovascular diseases.
Abbreviations: CI, confidence interval; G, group; eGFR, estimated glomerular filtration rate; eGFRcr, creatinine-based eGFR; eGFRcys, cystatin C-based eGFR; eGFRcr-cys, creatinine and cystatin C-based eGFR; MR-proADM, mid-regional pro-adrenomedullin; MR-proANP, mid-regional pro-atrial natriuretic peptide; NT-proBNP, N-terminal pro-B-type natriuretic peptide; Ref, reference; SD, standard deviation.
* $p < 0.05$, ** $p < 0.01$, *** $p < 0.001$.

Table S7. Cross-sectional associations of 3 myocardial stress markers with CKD [a]

Markers	CKD	Items	Categories of markers [b]				per 1 SD increase in log-transformed
			G1	G2	G3	G4	
**NT-proBNP**		Median, pg/ml	25.4	72.5	169.4	501.2	48.0
	**CKDcr**						
		Cases / controls (%)	539 / 30400 (1.8%)	543 / 20741 (2.6%)	364 / 6745 (5.4%)	387 / 2111 (18.3%)	1833 / 59997 (3.1%)
		Model 1	Ref.	1.11 (0.98, 1.26)	1.60 (1.38, 1.86) ***	4.30 (3.67, 5.05) ***	1.53 (1.45, 1.60) ***
		Model 2	Ref.	1.10 (0.97, 1.25)	1.51 (1.30, 1.76) ***	3.93 (3.33, 4.64) ***	1.49 (1.42, 1.57) ***
	**CKDcys**						
		Cases / controls (%)	878 / 30061 (2.9%)	1562 / 19722 (7.9%)	1310 / 5799 (22.6%)	913 / 1585 (57.6%)	4663 / 57167 (8.2%)
		Model 1	Ref.	1.54 (1.40, 1.69) ***	2.59 (2.33, 2.87) ***	4.91 (4.33, 5.57) ***	1.67 (1.61, 1.73) ***
		Model 2	Ref.	1.63 (1.49, 1.79) ***	2.72 (2.44, 3.04) ***	4.88 (4.28, 5.57) ***	1.68 (1.62, 1.75) ***
	**CKDcr-cys**						
		Cases / controls (%)	365 / 30574 (1.2%)	530 / 20754 (2.6%)	538 / 6571 (8.2%)	523 / 1975 (26.5%)	1956 / 59874 (3.3%)
		Model 1	Ref.	1.35 (1.17, 1.55) ***	2.62 (2.25, 3.06) ***	6.14 (5.22, 7.24) ***	1.84 (1.75, 1.93) ***
		Model 2	Ref.	1.37 (1.19, 1.58) ***	2.56 (2.19, 2.99) ***	5.72 (4.82, 6.78) ***	1.81 (1.72, 1.90) ***
**MR-proANP**		Median, pmol/l	30.8	53.7	92.8	147.2	46.8
	**CKDcr**						
		Cases / controls (%)	110 / 3430 (3.2%)	250 / 4243 (5.9%)	86 / 962 (8.9%)	77 / 341 (22.6%)	523 / 8976 (5.8%)
		Model 1	Ref.	1.17 (0.91, 1.51)	1.39 (1.00, 1.95)	3.30 (2.30, 4.74) ***	1.37 (1.24, 1.52) ***
		Model 2	Ref.	1.18 (0.92, 1.53)	1.35 (0.96, 1.89)	2.93 (2.01, 4.26) ***	1.31 (1.18, 1.45) ***
	**CKDcys**						
		Cases / controls (%)	19 / 3521 (0.5%)	119 / 4374 (2.7%)	97 / 951 (10.2%)	108 / 310 (34.8%)	343 / 9156 (3.7%)
		Model 1	Ref.	1.83 (1.13, 3.14) *	4.11 (2.46, 7.20) ***	10.4 (6.15, 18.4) ***	2.28 (2.01, 2.58) ***
		Model 2	Ref.	2.05 (1.26, 3.53) **	4.29 (2.54, 7.57) ***	9.87 (5.73, 17.7) ***	2.15 (1.89, 2.45) ***
	**CKDcr-cys**						
		Cases / controls (%)	39 / 3501 (1.1%)	124 / 4369 (2.8%)	58 / 990 (5.9%)	79 / 339 (23.3%)	300 / 9199 (3.3%)
		Model 1	Ref.	1.48 (1.00, 2.21)	2.22 (1.40, 3.57) ***	7.35 (4.63, 11.8) ***	1.92 (1.70, 2.18) ***
		Model 2	Ref.	1.54 (1.05, 2.31) *	2.17 (1.36, 3.50) **	6.39 (3.96, 10.5) ***	1.80 (1.58, 2.05) ***

Markers	CKD	Items	Categories of markers [b]				per 1 SD increase in log-transformed
			G1	G2	G3	G4	
**MR-proADM**		Median, nmol/l	0.37	0.50	0.66	0.86	0.46
	**CKDcr**						
		Cases / controls (%)	123 / 3369 (3.7%)	226 / 4171 (5.4%)	72 / 957 (7.5%)	98 / 311 (31.5%)	519 / 8808 (5.9%)
		Model 1	Ref.	1.21 (0.96, 1.53)	1.62 (1.16, 2.25) **	7.12 (5.09, 9.95) ***	1.72 (1.57, 1.90) ***
		Model 2	Ref.	1.25 (0.99, 1.60)	1.68 (1.19, 2.37) **	7.29 (5.05, 10.5) ***	1.73 (1.56, 1.92) ***
	**CKDcys**						
		Cases / controls (%)	11 / 3481 (0.3%)	85 / 4312 (2.0%)	100 / 929 (10.8%)	144 / 265 (54.3%)	340 / 8987 (3.8%)
		Model 1	Ref.	4.09 (2.26, 8.17) ***	20.8 (11.2, 42.5) ***	130.2 (68.3, 271.5) ***	5.33 (4.56, 6.27) ***
		Model 2	Ref.	3.96 (2.17, 7.96) ***	19.8 (10.4, 41.2) ***	130.4 (65.7, 280.9) ***	5.42 (4.57, 6.45) ***
	**CKDcr-cys**						
		Cases / controls (%)	32 / 3460 (0.9%)	98 / 4299 (2.3%)	58 / 971 (6.0%)	108 / 301 (35.9%)	296 / 9031 (3.3%)
		Model 1	Ref.	1.86 (1.25, 2.84) **	4.52 (2.83, 7.35) ***	28.8 (18.1, 46.8) ***	2.93 (2.59, 3.32) ***
		Model 2	Ref.	2.02 (1.35, 3.12) ***	5.04 (3.08, 8.36) ***	34.6 (20.8, 58.6) ***	3.04 (2.65, 3.48) ***

[a] Logistic regression was used to estimate OR and 95% CI of prevalent CKD for G2-4 compared with G1 of makers, as well as for per 1 SD increase in log-transformed markers.
[b] Categories of NT-proBNP: G1: < 48; G2: 48-125; G3: 125-300; G4: ≥ 300 pg/ml. Categories of MR-proANP: G1: < 40; G2: 40-80; G3: 80-120; G4: ≥ 120 pmol/l. Categories of MR-proADM: G1: < 0.425; G2: 0.425-0.609; G3: 0.609-0.766; G4: ≥ 0.766 nmol/l.
Model 1: adjusted for age, sex, and study cohort;
Model 2: model 1 plus body mass index, smoking status, alcohol consumption, systolic blood pressure, use of antihypertensive medication, high-density lipoprotein cholesterol, log-transformed triglycerides, diabetes, and cardiovascular diseases.
Abbreviations: CI, confidence interval; CKD, chronic kidney disease; CKDcr, creatinine-based CKD; CKDcys, cystatin C-based CKD; CKDcr-cys, creatinine and cystatin C-based CKD; G, group; MR-proADM, mid-regional pro-adrenomedullin; MR-proANP, mid-regional pro-atrial natriuretic peptide; NT-proBNP, N-terminal pro-B-type natriuretic peptide; OR, odds ratio; Ref, reference; SD, standard deviation.
* p < 0.05, ** p < 0.01, *** p < 0.001.

Table S8. Sensitivity analysis of cross-sectional associations of 3 myocardial stress markers with CKD [a]

Markers	Items	Categories of markers [b]				per 1 SD increase in log-transformed
		G1	G2	G3	G4	
**NT-proBNP**						
	Median, pg/ml	25.3	72.0	167.7	489.5	45.7
	Cases / controls (%)	154 / 29680 (0.5%)	219 / 19406 (1.1%)	230 / 5669 (4.1%)	294 / 1498 (19.6%)	897 / 56253 (1.6%)
	Model 2	Ref.	1.41 (1.14, 1.76) **	2.87 (2.27, 3.63) ***	9.22 (7.24, 11.8) ***	2.19 (2.04, 2.35) ***
**MR-proANP**						
	Median, pmol/l	30.8	53.5	92.8	146.3	45.9
	Cases / controls (%)	6 / 3417 (0.2%)	27 / 4152 (0.7%)	29 / 895 (3.2%)	50 / 286 (17.5%)	112 / 8750 (1.3%)
	Model 2	Ref.	1.41 (0.59, 3.91)	3.81 (1.55, 10.9) **	13.6 (5.53, 39.3) ***	2.75 (2.21, 3.44) ***
**MR-proADM**						
	Median, nmol/l	0.37	0.49	0.66	0.86	0.46
	Cases / controls (%)	3 / 3361 (0.1%)	17 / 4104 (0.4%)	19 / 876 (2.2%)	73 / 244 (29.9%)	112 / 8585 (1.3%)
	Model 2	Ref.	3.34 (1.10, 14.5)	17.6 (5.28, 80.6) ***	285.9 (85.7, 1331.4) ***	8.21 (6.17, 11.2) ***

[a] CKD cases were redefined as all 3 eGFR < 60 ml/min per 1.73m^2 and non-cases were redefined as all 3 eGFR ≥ 60 ml/min per 1.73m^2. Logistic regression was used to estimate OR and 95% CI of prevalent CKD for G2-4 compared with G1 of makers, as well as for per 1 SD increase in log-transformed markers.

[b] Categories of NT-proBNP: G1: < 48; G2: 48-125; G3: 125-300; G4: ≥ 300 pg/ml. Categories of MR-proANP: G1: < 40; G2: 40-80; G3: 80-120; G4: ≥ 120 pmol/l. Categories of MR-proADM: G1: < 0.425; G2: 0.425-0.609; G3: 0.609-0.766; G4: ≥ 0.766 nmol/l.

Model 2: adjusted for age, sex, study cohort, body mass index, smoking status, alcohol consumption, systolic blood pressure, use of antihypertensive medication, high-density lipoprotein cholesterol, log-transformed triglycerides, diabetes, and cardiovascular diseases.

Abbreviations: CI, confidence interval; CKD, chronic kidney disease; CKDcr, creatinine-based CKD; CKDcys, cystatin C-based CKD; CKDcr-cys, creatinine and cystatin C-based CKD; eGFR, estimated glomerular filtration rate; G, group; MR-proADM, mid-regional pro-adrenomedullin; MR-proANP, mid-regional pro-atrial natriuretic peptide; NT-proBNP, N-terminal pro-B-type natriuretic peptide; OR, odds ratio; Ref, reference; SD, standard deviation.

* $p < 0.05$, ** $p < 0.01$, *** $p < 0.001$.

Table S9. Longitudinal associations of NT-proBNP with incident CKD [a]

Incident CKD	Items	Categories of NT-proBNP [b]				per 1 SD increase in log-transformed
		G1	G2	G3	G4	
**Incident CKDcr**						
	Median, pg/ml	25.7	72.2	166.2	477.6	45.9
	Cases / person-year	59 / 24335	98 / 16289	54 / 4598	25 / 814	236 / 46036
	Model 1	Ref.	1.38 (1.02, 1.87) *	1.41 (0.99, 2.01)	3.91 (2.58, 5.92) ***	1.45 (1.28, 1.63) ***
	Model 2	Ref.	1.27 (0.94, 1.72)	1.13 (0.79, 1.61)	2.59 (1.66, 4.04) ***	1.26 (1.12, 1.42) ***
	Model 3	Ref.	1.09 (0.80, 1.48)	1.14 (0.79, 1.63)	1.86 (1.15, 3.01) *	1.16 (1.01, 1.32) *
**Incident CKDcys**						
	Median, pg/ml	25.2	72.4	162.5	503.1	45.9
	Cases / person-year	73 / 16118	109 / 10774	69 / 2933	22 / 456	273 / 30280
	Model 1	Ref.	1.22 (0.93, 1.60)	1.58 (1.16, 2.15) **	3.53 (2.25, 5.53) ***	1.44 (1.27, 1.62) ***
	Model 2	Ref.	1.22 (0.94, 1.59)	1.42 (1.04, 1.93) *	3.06 (1.95, 4.79) ***	1.33 (1.19, 1.49) ***
	Model 3	Ref.	1.16 (0.89, 1.52)	1.16 (0.83, 1.63)	1.87 (1.19, 2.95) **	1.17 (1.04, 1.32) **
**Incident CKDcr-cys**						
	Median, pg/ml	25.3	72.6	161.9	509.2	46.8
	Cases / person-year	40 / 16225	67 / 10954	50 / 3131	22 / 546	179 / 30856
	Model 1	Ref.	1.30 (0.92, 1.83)	1.72 (1.17, 2.52) **	5.31 (3.30, 8.55) ***	1.53 (1.33, 1.76) ***
	Model 2	Ref.	1.24 (0.87, 1.75)	1.50 (0.99, 2.29)	4.40 (2.63, 7.36) ***	1.39 (1.20, 1.62) ***
	Model 3	Ref.	1.01 (0.69, 1.49)	1.23 (0.81, 1.85)	2.59 (1.55, 4.34) ***	1.21 (1.02, 1.43) *

[a] Interval-censored Cox regression was used to estimate HR and 95% CI (1000 bootstrap samples for 95% CI estimation) of incident CKD for G2-4 compared with G1 of NT-proBNP, as well as for per 1 SD increase in log-transformed NT-proBNP. A total of 4167 participants free of CKDcr, 2557 free of CKDcys, and 2621 free of CKDcr-cys at baseline were included in these analyses.

[b] Categories of NT-proBNP: G1: < 48; G2: 48-125; G3: 125-300; G4: ≥ 300 pg/ml.

Please refer to the footnote of Table 4 for detailed information on Model 1, Model 2, and Model 3.

Abbreviations: CI, confidence interval; CKD, chronic kidney disease; CKDcr, creatinine-based CKD; CKDcys, cystatin C-based CKD; CKDcr-cys, creatinine and cystatin C-based CKD; G, group; HR, hazard ratio; NT-proBNP, N-terminal pro-B-type natriuretic peptide; Ref, reference; SD, standard deviation.

* $p < 0.05$, ** $p < 0.01$, *** $p < 0.001$.

Table S10. Cross-sectional associations of NT-proBNP with kidney function stratified by CVD and diabetes

Stratified factors	Items	Categories of NT-proBNP [b]				per 1 SD increase in log-transformed	P-interaction [a]
		G1	G2	G3	G4		
**Non-CVD**							
	Median, pg/ml	25.3	72.3	167.7	474.2	46.6	
	N	30411	20554	6401	1901	59267	
	eGFRcr	Ref.	-0.08 (-0.32, 0.17)	-0.97 (-1.35, -0.58) ***	-6.54 (-7.18, -5.90) ***	-0.87 (-0.99, -0.74) ***	< 0.001
	eGFRcys	Ref.	-2.02 (-2.33, -1.70) ***	-5.12 (-5.61, -4.63) ***	-11.0 (-11.8, -10.2) ***	-2.56 (-2.73, -2.40) ***	< 0.001
	eGFRcr-cys	Ref.	-1.40 (-1.68, -1.12) ***	-3.94 (-4.38, -3.51) ***	-10.5 (-11.2, -9.77) ***	-2.13 (-2.27, -1.98) ***	< 0.001
**CVD**							
	Median, pg/ml	27.4	81.0	187.9	579.7	128.7	
	N	528	730	708	597	2563	
	eGFRcr	Ref.	-1.09 (-2.88, 0.69)	-3.09 (-4.94, -1.23) **	-9.00 (-11.0, -7.02) ***	-3.13 (-3.66, -2.59) ***	
	eGFRcys	Ref.	-3.85 (-5.83, -1.88) ***	-7.76 (-9.81, -5.71) ***	-15.7 (-17.9, -13.5) ***	-5.09 (-5.68, -4.51) ***	
	eGFRcr-cys	Ref.	-2.93 (-4.82, -1.04) **	-6.53 (-8.50, -4.57) ***	-14.4 (-16.5, -12.3) ***	-4.76 (-5.32, -4.20) ***	
**Non-diabetes**							
	Median, pg/ml	25.3	72.4	168.9	486.4	47.2	
	N	29899	20329	6571	2141	58940	
	eGFRcr	Ref.	-0.06 (-0.31, 0.19)	-0.91 (-1.29, -0.53) ***	-6.37 (-6.99, -5.75) ***	-0.88 (-1.01, -0.75) ***	< 0.001
	eGFRcys	Ref.	-1.96 (-2.28, -1.65) ***	-5.00 (-5.48, -4.52) ***	-11.4 (-12.2, -10.6) ***	-2.63 (-2.79, -2.47) ***	< 0.001
	eGFRcr-cys	Ref.	-1.35 (-1.63, -1.07) ***	-3.83 (-4.26, -3.40) ***	-10.6 (-11.3, -9.93) ***	-2.17 (-2.31, -2.03) ***	< 0.001
**Diabetes**							
	Median, pg/ml	26.4	75.3	178.9	564.2	70.2	
	N	1040	955	538	357	2890	
	eGFRcr	Ref.	-0.94 (-2.34, 0.46)	-4.14 (-5.90, -2.39) ***	-11.1 (-13.2, -9.04) ***	-3.26 (-3.81, -2.71) ***	
	eGFRcys	Ref.	-3.97 (-5.91, -2.03) ***	-9.23 (-11.7, -6.80) ***	-16.3 (-19.1, -13.4) ***	-4.81 (-5.57, -4.05) ***	
	eGFRcr-cys	Ref.	-3.06 (-4.72, -1.39) ***	-8.11 (-10.2, -6.02) ***	-15.9 (-18.4, -13.5) ***	-4.67 (-5.33, -4.02) ***	

[a] Interaction terms of standardized log-transformed markers with CVD or diabetes were included in model 2 described in Table S6 to test the significance of interaction.
[b] Categories of NT-proBNP: G1: < 48; G2: 48-125; G3: 125-300; G4: ≥ 300 pg/ml.
Abbreviations: CVD, cardiovascular disease; G, group; eGFR, estimated glomerular filtration rate; eGFRcr, creatinine-based eGFR; eGFRcys, cystatin C-based eGFR; eGFRcr-cys, creatinine and cystatin C-based eGFR; NT-proBNP, N-terminal pro-B-type natriuretic peptide; Ref, reference; SD, standard deviation;
* $p < 0.05$, ** $p < 0.01$, *** $p < 0.001$.

Table S11. Cross-sectional associations of MR-proANP with kidney function stratified by CVD and diabetes

Stratified factors	Items	Categories of MR-proANP [b]				per 1 SD increase in log-transformed	P-interaction [a]
		G1	G2	G3	G4		
**Non-CVD**							
	Median, pmol/l	30.8	53.5	92.2	143.2	45.7	
	N	3469	4265	895	296	8925	
	eGFRcr	Ref.	-2.14 (-3.02, -1.26) ***	-2.36 (-3.81, -0.91) **	-6.04 (-8.24, -3.83) ***	-1.27 (-1.75, -0.80) ***	0.013
	eGFRcys	Ref.	-2.77 (-3.46, -2.09) ***	-6.68 (-7.81, -5.54) ***	-13.2 (-15.0, -11.5) ***	-3.33 (-3.70, -2.96) ***	< 0.001
	eGFRcr-cys	Ref.	-2.87 (-3.58, -2.15) ***	-5.46 (-6.64, -4.29) ***	-11.4 (-13.2, -9.65) ***	-2.76 (-3.15, -2.38) ***	< 0.001
**CVD**							
	Median, pmol/l	33.1	59.8	97.0	163.0	76.3	
	N	71	228	153	122	574	
	eGFRcr	Ref.	3.37 (-1.72, 8.47)	-2.36 (-7.93, 3.21)	-4.41 (-10.4, 1.57)	-2.62 (-4.10, -1.15) ***	
	eGFRcys	Ref.	0.66 (-3.73, 5.05)	-5.15 (-9.95, -0.35) *	-11.4 (-16.6, -6.25) ***	-4.43 (-5.70, -3.17) ***	
	eGFRcr-cys	Ref.	2.32 (-2.02, 6.66)	-4.36 (-9.10, 0.38)	-8.85 (-13.9, -3.75) ***	-3.98 (-5.23, -2.72) ***	
**Non-diabetes**							
	Median, pmol/l	30.8	53.6	92.7	147.0	46.5	
	N	3402	4278	966	369	9015	
	eGFRcr	Ref.	-2.07 (-2.96, -1.18) ***	-2.80 (-4.24, -1.37) ***	-5.87 (-7.94, -3.80) ***	-1.34 (-1.81, -0.87) ***	0.049
	eGFRcys	Ref.	-2.52 (-3.21, -1.82) ***	-6.41 (-7.53, -5.30) ***	-13.0 (-14.6, -11.4) ***	-3.29 (-3.65, -2.92) ***	< 0.001
	eGFRcr-cys	Ref.	-2.67 (-3.39, -1.95) ***	-5.54 (-6.70, -4.39) ***	-11.1 (-12.8, -9.47) ***	-2.76 (-3.14, -2.39) ***	< 0.001
**Diabetes**							
	Median, pmol/l	30.7	56.2	94.1	148.0	55.3	
	N	138	215	82	49	484	
	eGFRcr	Ref.	0.56 (-3.15, 4.26)	-1.68 (-6.71, 3.35)	-11.4 (-17.3, -5.39) ***	-2.67 (-4.16, -1.19) ***	
	eGFRcys	Ref.	-5.19 (-8.89, -1.49) **	-12.3 (-17.3, -7.27) ***	-21.1 (-27.1, -15.2) ***	-5.95 (-7.41, -4.49) ***	
	eGFRcr-cys	Ref.	-2.95 (-6.45, 0.56)	-8.27 (-13.0, -3.52) ***	-18.2 (-23.8, -12.5) ***	-4.92 (-6.31, -3.54) ***	

[a] Interaction terms of standardized log-transformed markers with CVD or diabetes were included in model 2 described in Table S6 to test the significance of interaction.
[b] Categories of MR-proANP: G1: < 40; G2: 40-80; G3: 80-120; G4: ≥ 120 pmol/l.
Abbreviations: CVD, cardiovascular disease; G, group; eGFR, estimated glomerular filtration rate; eGFRcr, creatinine-based eGFR; eGFRcys, cystatin C-based eGFR; eGFRcr-cys, creatinine and cystatin C-based eGFR; MR-proANP, mid-regional pro-atrial natriuretic peptide; Ref, reference; SD, standard deviation;
* $p < 0.05$, ** $p < 0.01$, *** $p < 0.001$.

Table S12. Cross-sectional associations of MR-proADM with kidney function stratified by CVD and diabetes

Stratified factors	Items	Categories of MR-proADM [b]				per 1 SD increase in log-transformed	P-interaction [a]
		G1	G2	G3	G4		
**Non-CVD**							
	Median, nmol/l	0.37	0.49	0.66	0.86	0.46	
	N	3410	4130	892	326	8758	
	eGFRcr	Ref.	-2.74 (-3.59, -1.88) ***	-4.82 (-6.26, -3.37) ***	-10.1 (-12.3, -8.01) ***	-2.68 (-3.14, -2.23) ***	0.004
	eGFRcys	Ref.	-6.29 (-6.93, -5.66) ***	-14.4 (-15.5, -13.3) ***	-24.9 (-26.5, -23.3) ***	-6.57 (-6.91, -6.23) ***	< 0.001
	eGFRcr-cys	Ref.	-5.22 (-5.89, -4.55) ***	-11.4 (-12.5, -10.2) ***	-20.3 (-22.0, -18.6) ***	-5.38 (-5.74, -5.02) ***	< 0.001
**CVD**							
	Median, nmol/l	0.39	0.52	0.68	0.86	0.57	
	N	82	267	137	83	569	
	eGFRcr	Ref.	-5.93 (-10.4, -1.47) **	-8.50 (-13.7, -3.26) **	-18.4 (-24.5, -12.3) ***	-5.18 (-6.82, -3.55) ***	
	eGFRcys	Ref.	-5.62 (-9.21, -2.02) **	-17.5 (-21.7, -13.3) ***	-26.0 (-30.9, -21.1) ***	-9.04 (-10.3, -7.75) ***	
	eGFRcr-cys	Ref.	-6.58 (-10.2, -2.96) ***	-15.1 (-19.3, -10.8) ***	-25.4 (-30.4, -20.5) ***	-8.17 (-9.47, -6.87) ***	
**Non-diabetes**							
	Median, nmol/l	0.37	0.49	0.66	0.86	0.46	
	N	3386	4185	938	342	8851	
	eGFRcr	Ref.	-2.72 (-3.58, -1.87) ***	-4.85 (-6.28, -3.42) ***	-10.2 (-12.3, -8.08) ***	-2.65 (-3.11, -2.20) ***	0.002
	eGFRcys	Ref.	-6.16 (-6.79, -5.52) ***	-14.7 (-15.7, -13.6) ***	-24.6 (-26.1, -23.0) ***	-6.57 (-6.90, -6.23) ***	< 0.001
	eGFRcr-cys	Ref.	-5.13 (-5.80, -4.46) ***	-11.5 (-12.6, -10.4) ***	-20.2 (-21.8, -18.5) ***	-5.36 (-5.72, -5.01) ***	< 0.001
**Diabetes**							
	Median, nmol/l	0.36	0.51	0.68	0.87	0.53	
	N	106	212	91	67	476	
	eGFRcr	Ref.	-6.21 (-10.2, -2.17) **	-8.40 (-13.6, -3.23) **	-19.9 (-25.6, -14.1) ***	-6.16 (-7.65, -4.67) ***	
	eGFRcys	Ref.	-8.32 (-12.2, -4.50) ***	-17.8 (-22.7, -12.9) ***	-30.8 (-36.2, -25.3) ***	-9.68 (-11.1, -8.31) ***	
	eGFRcr-cys	Ref.	-8.35 (-12.0, -4.71) ***	-15.4 (-20.0, -10.7) ***	-28.7 (-33.8, -23.5) ***	-8.99 (-10.3, -7.68) ***	

[a] Interaction terms of standardized log-transformed markers with CVD or diabetes were included in model 2 described in Table S6 to test the significance of interaction.
[b] Categories of MR-proADM: G1: < 0.425; G2: 0.425-0.609; G3: 0.609-0.766; G4: ≥ 0.766 nmol/l.
Abbreviations: CVD, cardiovascular disease; G, group; eGFR, estimated glomerular filtration rate; eGFRcr, creatinine-based eGFR; eGFRcys, cystatin C-based eGFR; eGFRcr-cys, creatinine and cystatin C-based eGFR; MR-proADM, mid-regional pro-adrenomedullin; Ref, reference; SD, standard deviation;
* $p < 0.05$, ** $p < 0.01$, *** $p < 0.001$.

Table S13. Sensitivity analysis of cross-sectional associations of 3 myocardial stress markers with eGFRcr-cys and CKDcr-cys [a]

eGFR / CKD	Markers	Categories of markers [b]				per 1 SD increase in log-transformed
		G1	G2	G3	G4	
**eGFRcr-cys**						
		**Beta coefficient (95% CI)**				
	NT-proBNP	Ref.	-1.51 (-1.79, -1.24) ***	-4.31 (-4.73, -3.89) ***	-11.5 (-12.1, -10.8) ***	-2.44 (-2.58, -2.30) ***
	MR-proANP	Ref.	-2.68 (-3.38, -1.97) ***	-5.72 (-6.85, -4.60) ***	-11.8 (-13.4, -10.2) ***	-2.93 (-3.29, -2.56) ***
	MR-proADM	Ref.	-5.24 (-5.90, -4.58) ***	-11.6 (-12.7, -10.6) ***	-21.0 (-22.6, -19.5) ***	-5.60 (-5.94, -5.25) ***
**CKDcr-cys**						
		**Odds ratio (95% CI)**				
	NT-proBNP	Ref.	1.38 (1.19, 1.59) ***	2.58 (2.21, 3.01) ***	5.77 (4.87, 6.85) ***	1.81 (1.72, 1.90) ***
	MR-proANP	Ref.	1.54 (1.05, 2.31) *	2.17 (1.36, 3.50) **	6.40 (3.96, 10.5) ***	1.80 (1.58, 2.05) ***
	MR-proADM	Ref.	2.02 (1.35, 3.11) ***	5.04 (3.08, 8.36) ***	34.6 (20.8, 58.7) ***	3.04 (2.66, 3.49) ***

[a] Sensitivity analyses were conducted to test the robustness of the results by further adjusting for low-density lipoprotein cholesterol in model 2, as described in Table 2 and Table 3. These sensitivity analyses were limited to eGFRcr-cys and CKDcr-cys. Data from 61830 participants for NT-proBNP, 9499 for MR-proANP, and 9327 for MR-proADM were included in these analyses.

[b] Categories of NT-proBNP: G1: < 48; G2: 48-125; G3: 125-300; G4: ≥ 300 pg/ml. Categories of MR-proANP: G1: < 40; G2: 40-80; G3: 80-120; G4: ≥ 120 pmol/l. Categories of MR-proADM: G1: < 0.425; G2: 0.425-0.609; G3: 0.609-0.766; G4: ≥ 0.766 nmol/l.

Abbreviations: CI, confidence interval; CKDcr-cys, creatinine and cystatin C-based chronic kidney disease; G, group; eGFRcr-cys, creatinine and cystatin C-based estimated glomerular filtration rate; MR-proADM, mid-regional pro-adrenomedullin; MR-proANP, mid-regional pro-atrial natriuretic peptide; NT-proBNP, N-terminal pro-B-type natriuretic peptide; Ref, reference; SD, standard deviation.

* $p < 0.05$, ** $p < 0.01$, *** $p < 0.001$.

Table S14. Sensitivity analysis of longitudinal associations of NT-proBNP with change in eGFRcr-cys and incident CKDcr-cys [a]

eGFR / CKD	Items	Categories of NT-proBNP [b]				per 1 SD increase in log-transformed
		G1	G2	G3	G4	
**Change in eGFRcr-cys**						
		**Beta coefficient (95% CI)**				
	Model 2 + LDL-C	Ref.	-1.05 (-1.88, -0.21) *	-3.24 (-4.53, -1.94) ***	-7.02 (-9.66, -4.39) ***	-1.33 (-1.73, -0.93) ***
	Model 3 + LDL-C	Ref.	-1.14 (-2.07, -0.20) *	-3.55 (-4.98, -2.12) ***	-7.97 (-10.9, -5.08) ***	-1.50 (-1.94, -1.06) ***
**Incident CKDcr-cys**						
		**Hazard ratio (95% CI)**				
	Model 2 + LDL-C	Ref.	1.18 (0.83, 1.68)	1.43 (0.93, 2.19)	4.40 (2.66, 7.29) ***	1.39 (1.20, 1.61) ***
	Model 3 + LDL-C	Ref.	0.98 (0.68, 1.42)	1.17 (0.75, 1.83)	2.60 (1.52, 4.47) ***	1.21 (1.03, 1.42) *

[a] Sensitivity analyses were conducted to test the robustness of the results by further adjusting for low-density lipoprotein cholesterol in model 2 and model 3, as described in Table 4 and Table S9. These sensitivity analyses were limited to the 10-year change in eGFRcr-cys and incident CKDcr-cys. A total of 2651 participants with 7061 observations were included for the analysis with change in eGFRcr-cys and 2621 free of CKDcr-cys at baseline were included for the analysis with incident CKDcr-cys.

[b] Categories of NT-proBNP: G1: < 48; G2: 48-125; G3: 125-300; G4: ≥ 300 pg/ml.

Abbreviations: CI, confidence interval; CKDcr-cys, creatinine and cystatin C-based chronic kidney disease; G, group; eGFRcr-cys, creatinine and cystatin C-based estimated glomerular filtration rate; LDL-C, low-density lipoprotein cholesterol; NT-proBNP, N-terminal pro-B-type natriuretic peptide; Ref, reference; SD, standard deviation.

* $p < 0.05$, ** $p < 0.01$, *** $p < 0.001$.

Table S15. E-values to assess the robustness of observed associations to potential unmeasured or uncontrolled confounders [a]

eGFR / CKD	Markers	Cases / controls (%)	per 1 SD increase in log-transformed	E-values [b] Point estimate	Lower limit
**Prevalent CKDcr-cys**					
			**OR (95% CI)**	**OR**	
	NT-proBNP	1956 / 59874 (3.3%)	1.81 (1.72, 1.90) ***	3.02	2.83
	MR-proANP	300 / 9199 (3.3%)	1.80 (1.58, 2.05) ***	3.00	2.54
	MR-proADM	296 / 9031 (3.3%)	3.04 (2.65, 3.48) ***	5.53	4.74
**Incident CKDcr-cys**					
			**HR (95% CI)**	**HR**	
	NT-proBNP	179 / 2621	1.39 (1.20, 1.62) ***	2.13	1.69

[a] To assess the robustness of associations observed in our analyses to potential unmeasured or uncontrolled confounding, E-values were calculated [11, 12]. The E-value is defined as the minimum strength of association on the risk ratio scale that an unmeasured confounder would need to have with both exposure and outcome to fully explain away a specific exposure-outcome association, conditional on the measured covariates. In simpler terms, the E-value indicates how strongly an unmeasured confounder would have to be to fully account for the observed association. A large E-value (e.g., > 2) implies that considerable unmeasured confounding would be needed to explain away an effect estimate. In contrast, a small E-value implies that little unmeasured confounding would be needed to explain away an effect estimate.

[b] Results of per 1 SD increase in log-transformed markers based on model 2 as described in Table 2 and Table S9 were used to calculate E-values. E-value (point estimate) was calculated using the estimated OR (e.g., 1.81 for NT-proBNP with prevalent CKDcr-cys) based on the formula: Point estimate = OR + sqrt (OR * (OR - 1)). Similarly, E-value (lower limit) was calculated based on the same formula using the lower limit of 95% CI. The E-value for HR was calculated in a similar way as for the OR. The minimum point estimate is 2.13 in these calculations, which means that an unmeasured confounder would need to be associated with both exposure and outcome by a risk ratio of more than 2.13-fold each, above and beyond the measured confounders, to explain away the observed association. These E-values suggest that our results were robust to potential unmeasured confounding.

Abbreviations: CI, confidence interval; CKDcr-cys, creatinine and cystatin C-based chronic kidney disease; HR, hazard ratio; MR-proADM, mid-regional pro-adrenomedullin; MR-proANP, mid-regional pro-atrial natriuretic peptide; NT-proBNP, N-terminal pro-B-type natriuretic peptide; OR, odds ratio; Ref, reference; SD, standard deviation.

* $p < 0.05$, ** $p < 0.01$, *** $p < 0.001$.

Supplementary figures

Figure S1. Flowchart of study participants from the MONICA/KORA study included in the longitudinal analysis. The MONICA/KORA S3 baseline examination was carried out in 1994-1995 and participants were followed up after 10 years (2004-2005, F3). The KORA S4 baseline examination was carried out in 1999-2001, and participants were followed up after 7 years (2006-2008) and after 14 years (2013-2014).

Abbreviations: CKD, chronic kidney disease; cr, creatinine-based; cys, cystatin C-based; cr-cys, combined creatinine and cystatin C-based; eGFR, estimated glomerular filtration rate; KORA, Cooperative Health Research in the Region of Augsburg; MONICA, Monitoring of Trends and Determinants in Cardiovascular Diseases; NT-proBNP, N-terminal pro-B-type natriuretic peptide.

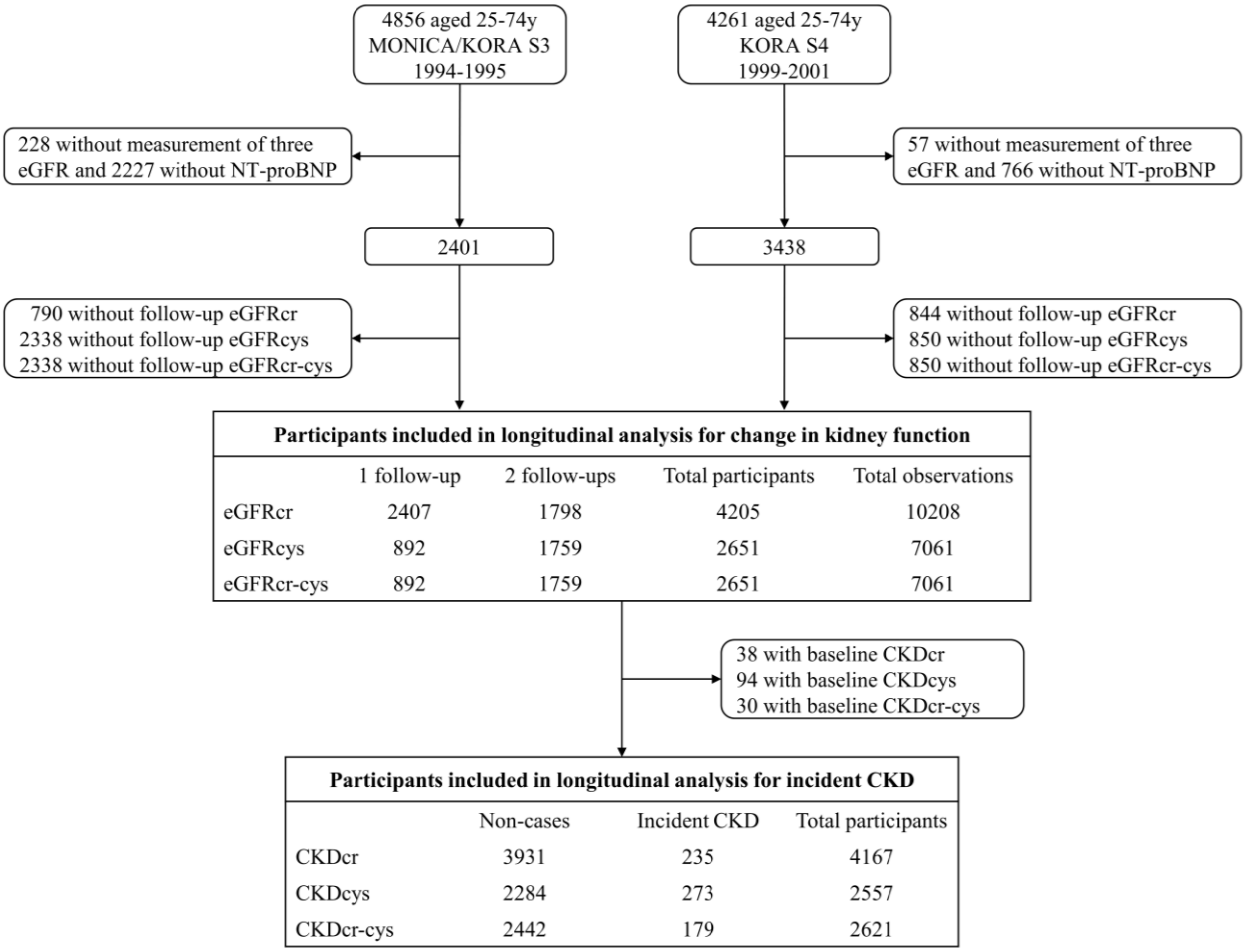

Figure S2. Cross-sectional associations of 3 myocardial stress markers with kidney function. Linear regression was used to estimate beta coefficients and 95% CI of eGFR across G2-4 compared with G1 of markers. Detailed information on adjusted models is described in Table 2. Data from 61830 participants for NT-proBNP, 9499 for MR-proANP, and 9327 for MR-proADM were included in these analyses. Categories of NT-proBNP: G1: < 48; G2: 48-125; G3: 125-300; G4: ≥ 300 pg/ml. Categories of MR-proANP: G1: < 40; G2: 40-80; G3: 80-120; G4: ≥ 120 pmol/l. Categories of MR-proADM: G1: < 0.425; G2: 0.425-0.609; G3: 0.609-0.766; G4: ≥ 0.766 nmol/l.

Abbreviations: CI, confidence interval; eGFR, estimated glomerular filtration rate; eGFRcr, creatinine-based eGFR; eGFRcys, cystatin C-based eGFR; eGFRcr-cys, creatinine and cystatin C-based eGFR; G, group; MR-proADM, mid-regional pro-adrenomedullin; MR-proANP, mid-regional pro-atrial natriuretic peptide; NT-proBNP, N-terminal pro-B-type natriuretic peptide; * $p < 0.05$, ** $p < 0.01$, *** $p < 0.001$.

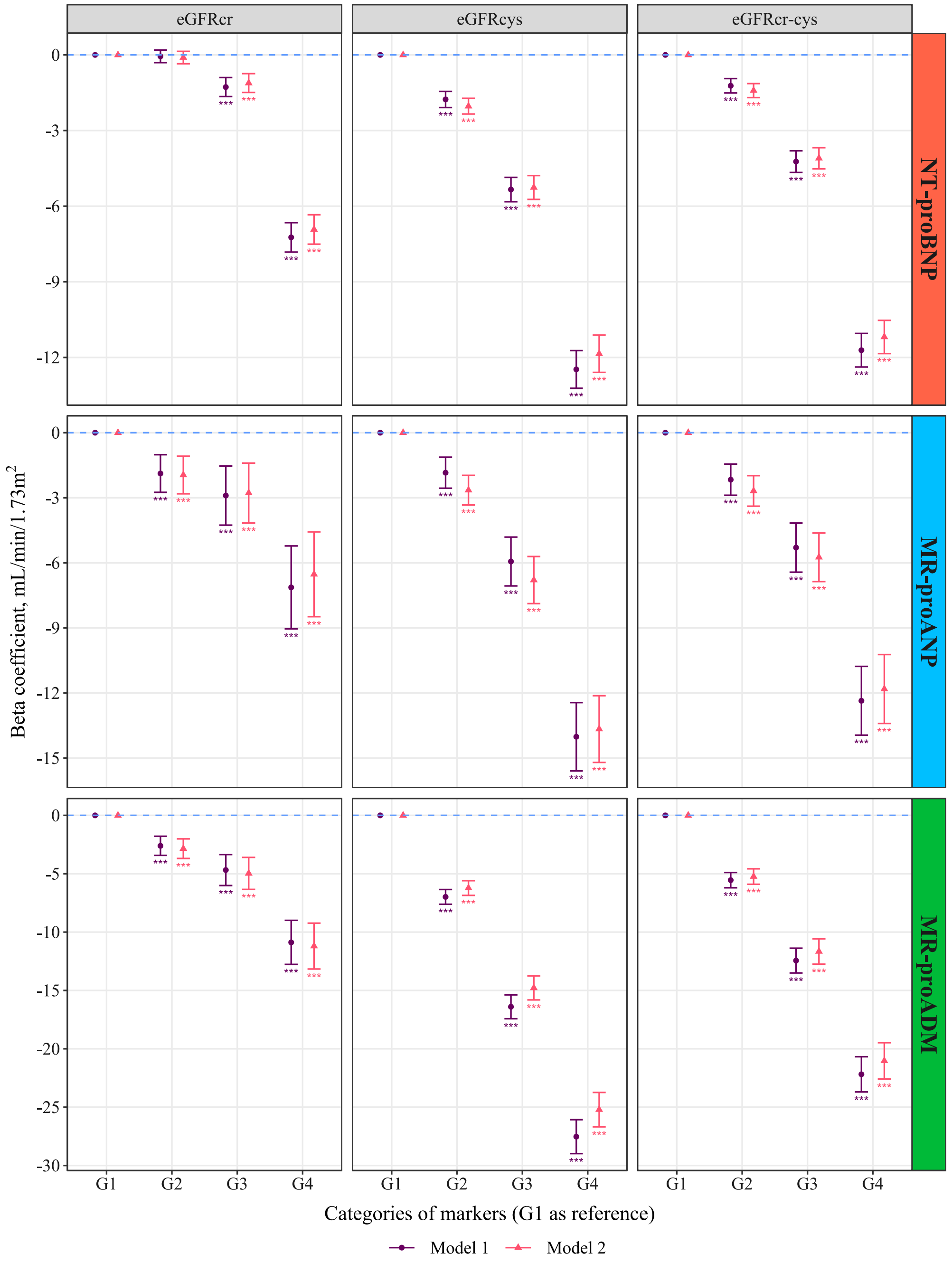

Figure S3. Cross-sectional associations of 3 myocardial stress markers with CKD. Logistic regression was used to estimate odds ratios and 95% CI of prevalent CKD across G2-4 compared with G1 of markers. Detailed information on adjusted models is described in Table 3. Data from 61830 participants for NT-proBNP, 9499 for MR-proANP, and 9327 for MR-proADM were included in these analyses. Categories of NT-proBNP: G1: < 48; G2: 48-125; G3: 125-300; G4: ≥ 300 pg/ml. Categories of MR-proANP: G1: < 40; G2: 40-80; G3: 80-120; G4: ≥ 120 pmol/l. Categories of MR-proADM: G1: < 0.425; G2: 0.425-0.609; G3: 0.609-0.766; G4: ≥ 0.766 nmol/l.

Abbreviations: CI, confidence interval; CKD, chronic kidney disease; CKDcr, creatinine-based CKD; CKDcys, cystatin C-based CKD; CKDcr-cys, creatinine and cystatin C-based CKD; G, group; MR-proADM, mid-regional pro-adrenomedullin; MR-proANP, mid-regional pro-atrial natriuretic peptide; NT-proBNP, N-terminal pro-B-type natriuretic peptide; * $p < 0.05$, ** $p < 0.01$, *** $p < 0.001$.

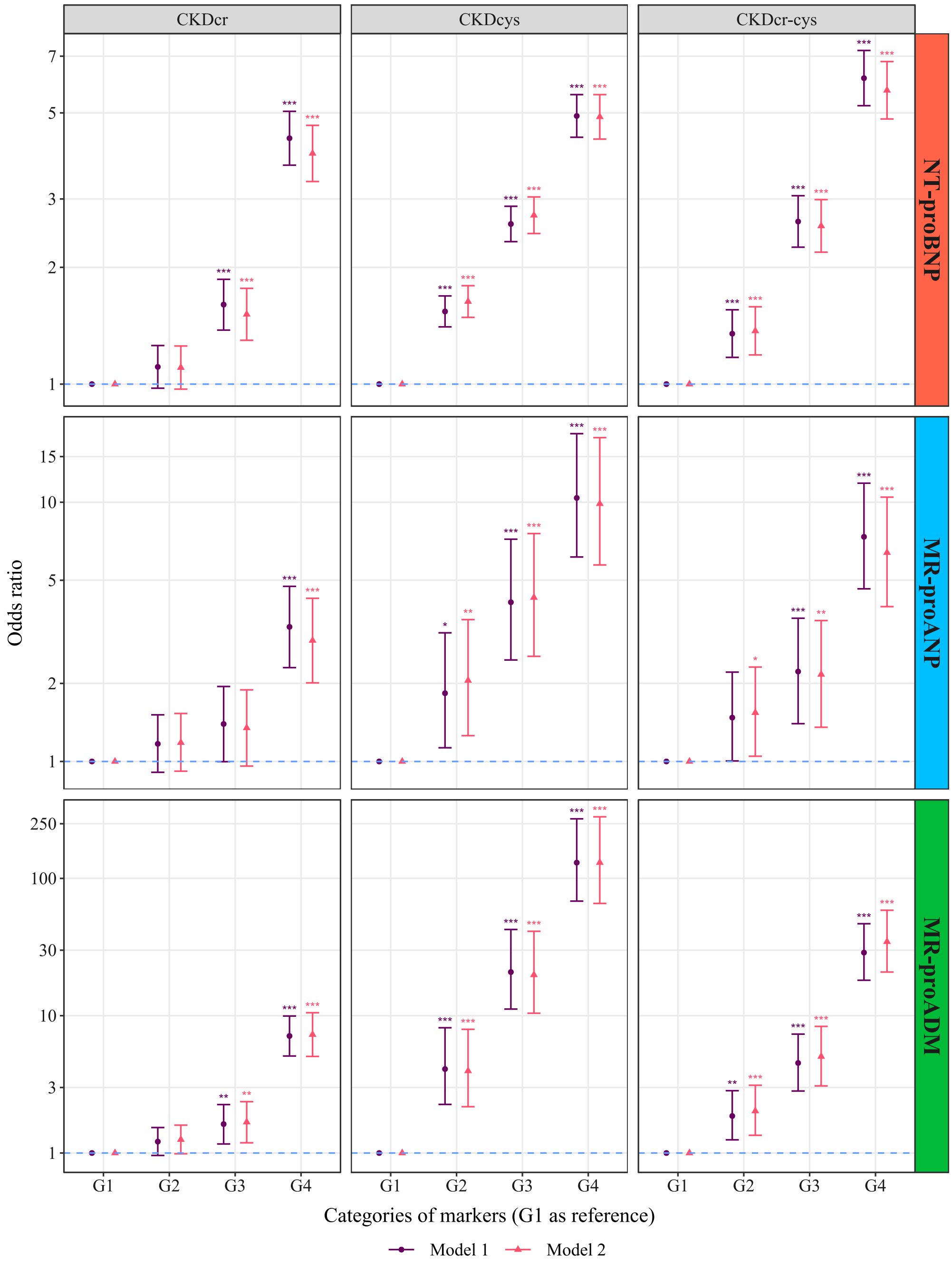

Figure S4. Sensitivity analysis of cross-sectional associations of 3 myocardial stress markers with CKD. CKD cases were redefined as all 3 eGFR < 60 ml/min per $1.73m^2$ and non-cases were redefined as all 3 eGFR ≥ 60 ml/min per $1.73m^2$. Logistic regression was used to estimate odds ratio and 95% CI of prevalent CKD per 1 SD increase in log-transformed markers. Detailed information on adjusted models is described in Table S8. Data from 57150 participants for NT-proBNP, 8862 for MR-proANP, and 8697 for MR-proADM were included in these analyses.

Abbreviations: CI, confidence interval; CKD, chronic kidney disease; CKDcr, creatinine-based CKD; CKDcys, cystatin C-based CKD; CKDcr-cys, creatinine and cystatin C-based CKD; eGFR, estimated glomerular filtration rate; MR-proADM, mid-regional pro-adrenomedullin; MR-proANP, mid-regional pro-atrial natriuretic peptide; NT-proBNP, N-terminal pro-B-type natriuretic peptide; SD, standard deviation; * $p < 0.05$, ** $p < 0.01$, *** $p < 0.001$.

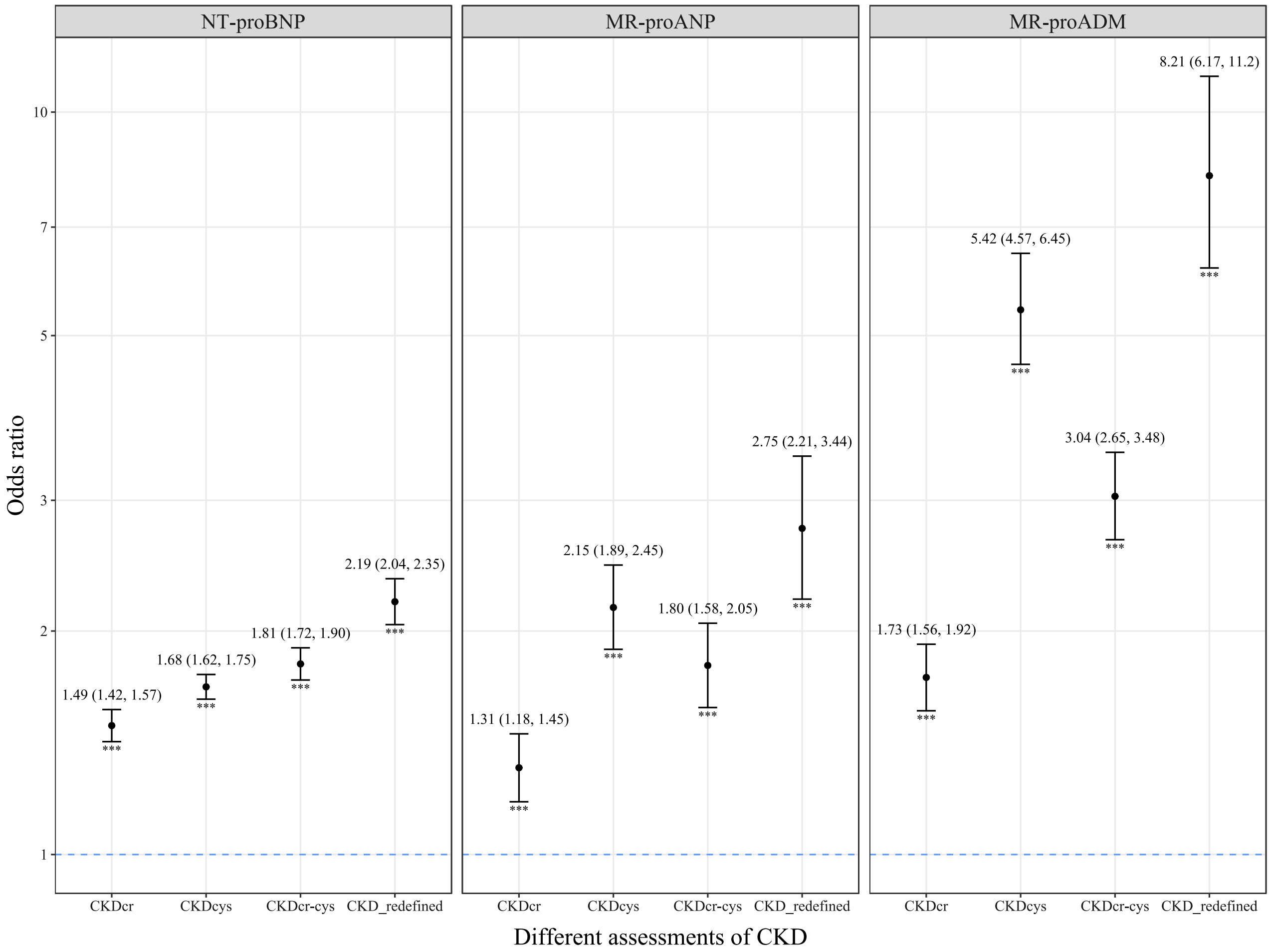

Figure S5. Cross-sectional associations of 3 myocardial stress markers with CKD stratified by CVD and diabetes. Interaction terms of standardized log-transformed markers with CVD or diabetes were included in logistic regression, applying model 2 described in Table 3, to test the significance of interaction. Data from 61830 participants for NT-proBNP, 9499 for MR-proANP, and 9327 for MR-proADM were included in these analyses.

Abbreviations: CVD, cardiovascular disease; CKD, chronic kidney disease; CKDcr, creatinine-based CKD; CKDcys, cystatin C-based CKD; CKDcr-cys, creatinine and cystatin C-based CKD; MR-proADM, mid-regional pro-adrenomedullin; MR-proANP, mid-regional pro-atrial natriuretic peptide; NT-proBNP, N-terminal pro-B-type natriuretic peptide; SD, standard deviation; * $p < 0.05$, ** $p < 0.01$, *** $p < 0.001$.

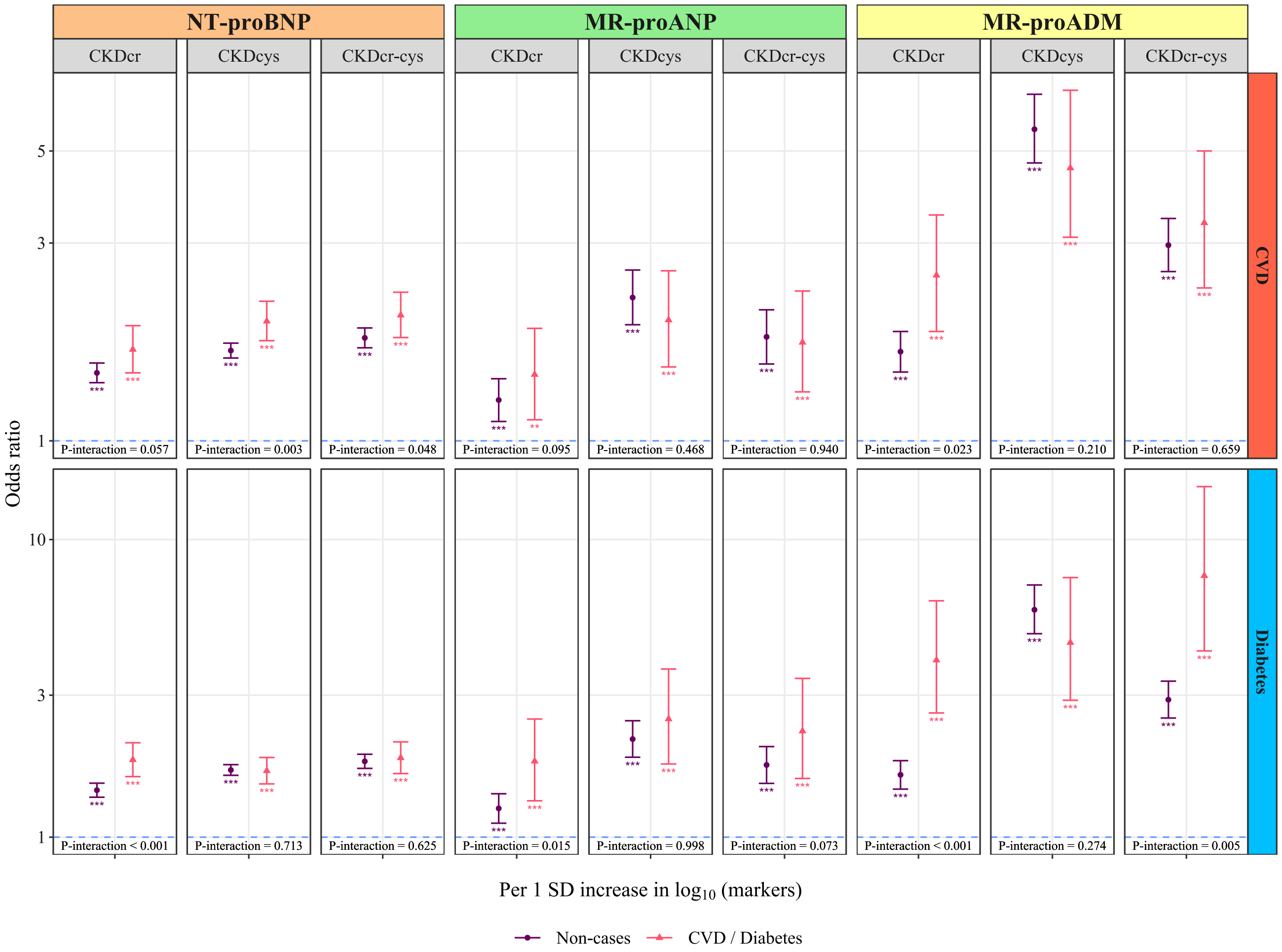

Figure S6. Longitudinal associations of NT-proBNP with 10-year change in kidney function and incident CKD stratified by CVD and diabetes. Interaction terms of standardized log-transformed markers with CVD or diabetes were included in model 3 described in Table 4 and Table S9 to test the significance of interaction. A maximum of 4205 participants with 10208 observations were included for analyses with change in kidney function and a maximum of 4167 participants free of CKDcr at baseline were included for analyses with incident CKD.

Abbreviations: CVD, cardiovascular disease; CKD, chronic kidney disease; cr, creatinine-based; cys, cystatin C-based; cr-cys, combined creatinine and cystatin C-based; eGFR, estimated glomerular filtration rate; NT-proBNP, N-terminal pro-B-type natriuretic peptide; SD, standard deviation; * $p < 0.05$, ** $p < 0.01$, *** $p < 0.001$.

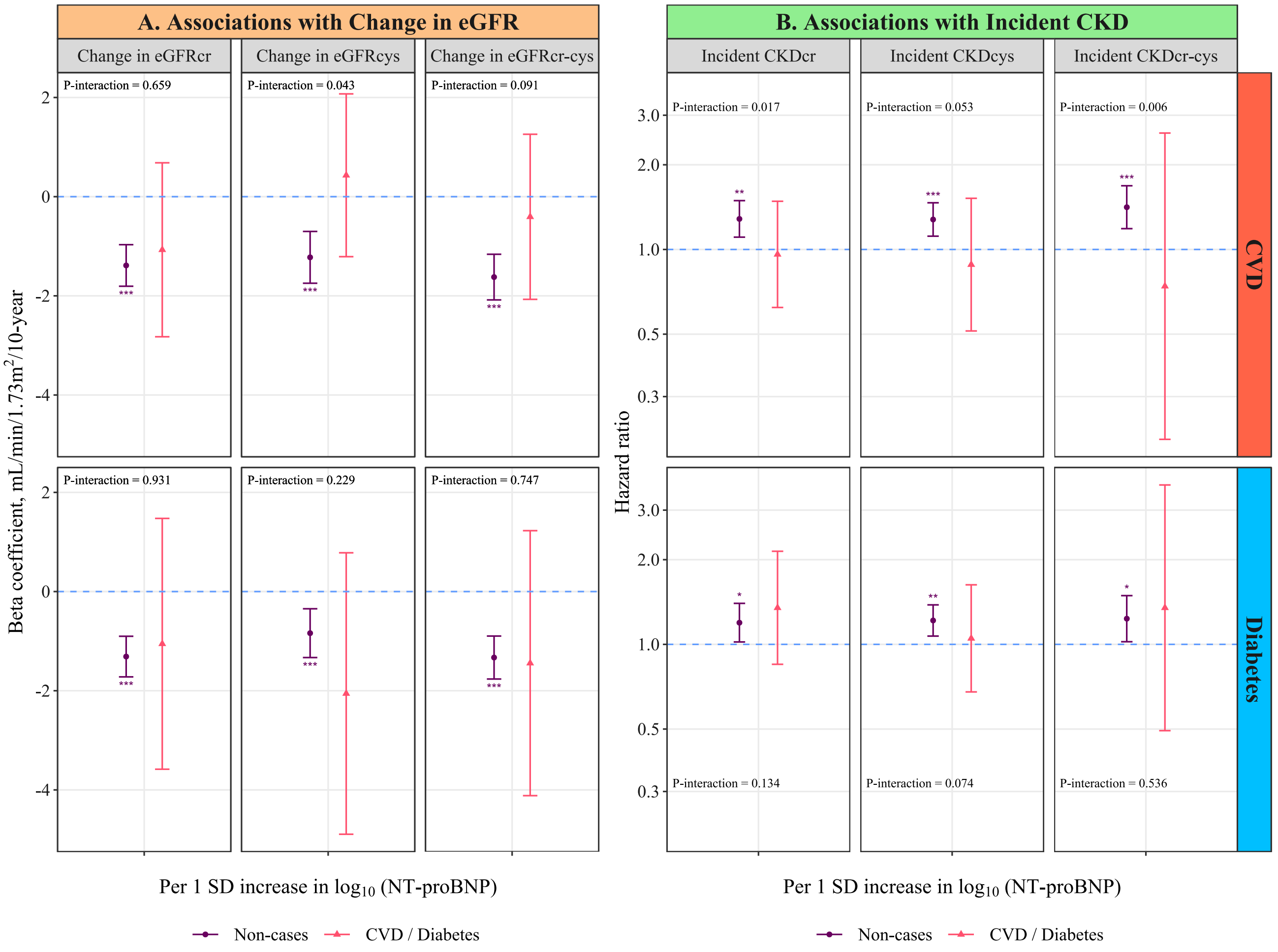

Figure S7. Shapes of the associations between 3 myocardial stress markers and prevalent CKDcr-cys. Restricted cubic spline functions were used to estimate the non-linear associations between original levels of markers and prevalent CKDcr-cys in logistic regression models, applying model 2 as described in Table 3. Participants with levels of markers < 2.5th percentile or > 97.5th percentile were not included in the non-linear association analysis. Finally, 58638 participants for NT-proBNP, 9030 for MR-proANP, and 8861 for MR-proADM were included in these analyses. ORs and 95% CI s of prevalent CKDcr-cys across G2-G4 compared with G1 of markers are plotted, using the minimum marker levels within each category as x-axis locations.

Abbreviations: CI, confidence interval; CKDcr-cys, creatinine and cystatin C-based CKD; G, group; MR-proADM, mid-regional pro-adrenomedullin; MR-proANP, mid-regional pro-atrial natriuretic peptide; NT-proBNP, N-terminal pro-B-type natriuretic peptide; OR, odds ratio.

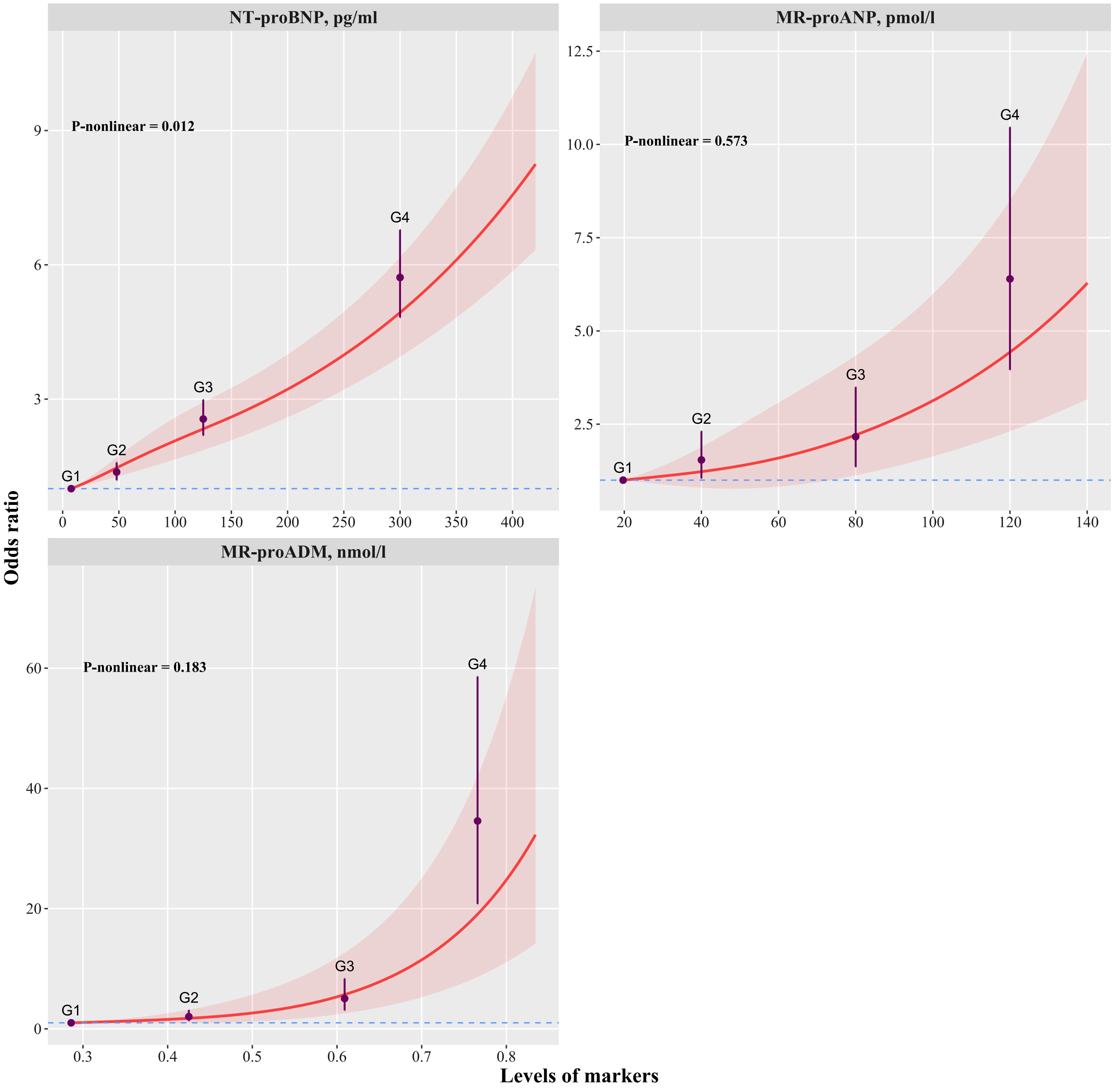

Figure S8. Shapes of the associations between NT-proBNP and incident CKD. Restricted cubic spline functions were used to estimate the non-linear associations between original levels of NT-proBNP and incident CKD in the Cox proportional hazards models, applying model 3 as described in Table 4. Participants with levels of NT-proBNP < 2.5th percentile or > 97.5th percentile were not included in the non-linear association analysis. Finally, 4013 participants free of CKDcr, 2465 free of CKDcys, and 2523 free of CKDcr-cys at baseline were included in these analyses. HRs and 95% CI s of incident CKD across G2-G4 compared with G1 of NT-proBNP are plotted, using the minimum NT-proBNP levels within each category as x-axis locations.

Abbreviations: CI, confidence interval; CKD, chronic kidney disease; CKDcr, creatinine-based CKD; CKDcys, cystatin C-based CKD; CKDcr-cys, creatinine and cystatin C-based CKD; G, group; HR, hazard ratio; NT-proBNP, N-terminal pro-B-type natriuretic peptide; SD, standard deviation.

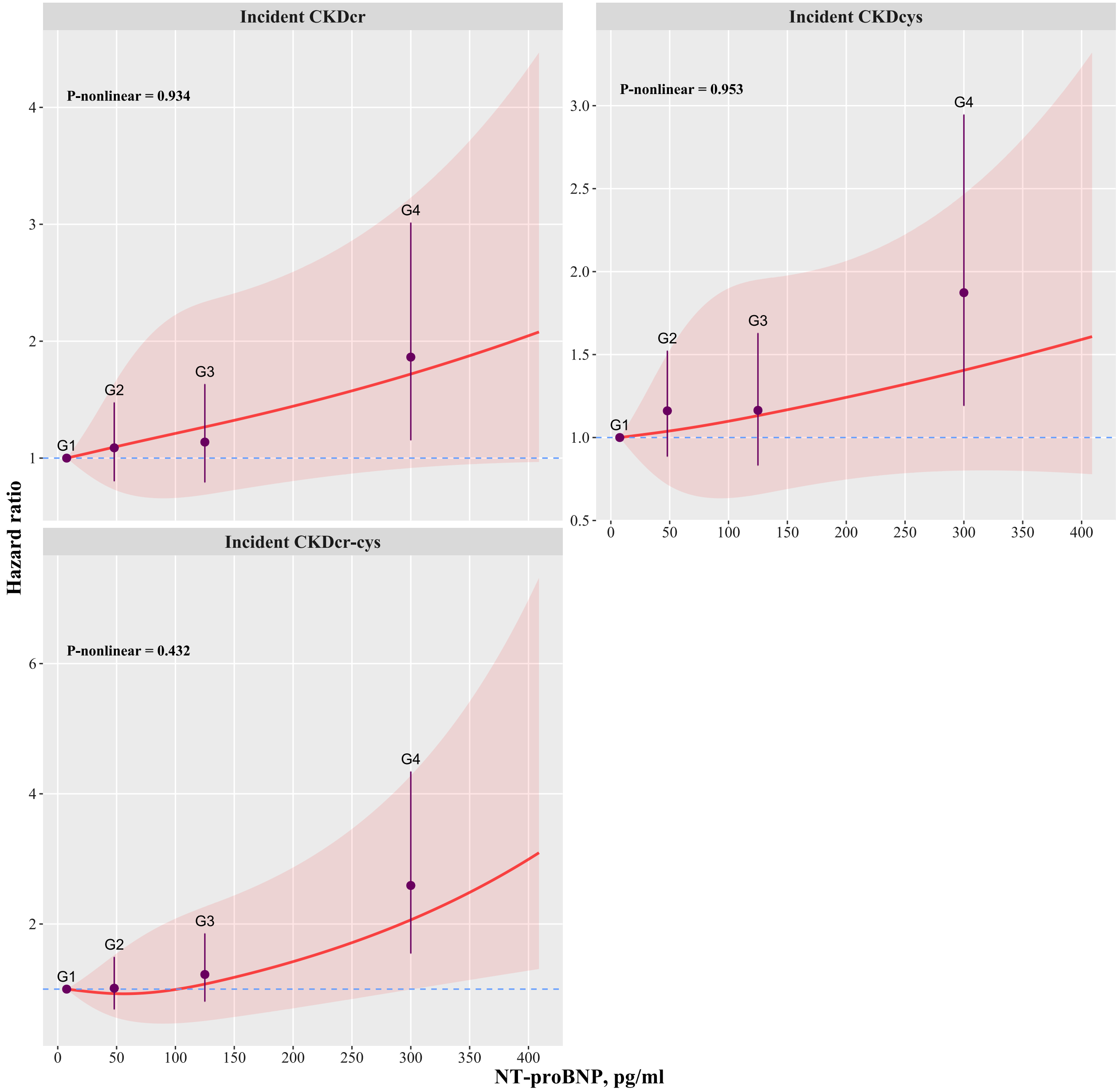

References

1. Inker LA, Eneanya ND, Coresh J*, et al.* New Creatinine- and Cystatin C-Based Equations to Estimate GFR without Race. *N Engl J Med* 2021; 385: 1737-1749.

2. Inker LA, Schmid CH, Tighiouart H*, et al.* Estimating glomerular filtration rate from serum creatinine and cystatin C. *N Engl J Med* 2012; 367: 20-29.

3. Borodulin K, Tolonen H, Jousilahti P*, et al.* Cohort Profile: The National FINRISK Study. *Int J Epidemiol* 2018; 47: 696-696i.

4. Lowel H, Doring A, Schneider A*, et al.* The MONICA Augsburg surveys--basis for prospective cohort studies. *Gesundheitswesen* 2005; 67 Suppl 1: S13-18.

5. Holle R, Happich M, Lowel H*, et al.* KORA--a research platform for population based health research. *Gesundheitswesen* 2005; 67 Suppl 1: S19-25.

6. Di Castelnuovo A, Costanzo S, Persichillo M*, et al.* Distribution of short and lifetime risks for cardiovascular disease in Italians. *Eur J Prev Cardiol* 2012; 19: 723-730.

7. Gianfagna F, Veronesi G, Guasti L*, et al.* Do apolipoproteins improve coronary risk prediction in subjects with metabolic syndrome? Insights from the North Italian Brianza cohort study. *Atherosclerosis* 2014; 236: 175-181.

8. Eriksson M, Holmgren L, Janlert U*, et al.* Large improvements in major cardiovascular risk factors in the population of northern Sweden: the MONICA study 1986-2009. *J Intern Med* 2011; 269: 219-231.

9. Yarnell JW. The PRIME study: classical risk factors do not explain the severalfold differences in risk of coronary heart disease between France and Northern Ireland. Prospective Epidemiological Study of Myocardial Infarction. *QJM* 1998; 91: 667-676.

10. Tunstall-Pedoe H, Woodward M, Tavendale R*, et al.* Comparison of the prediction by 27 different factors of coronary heart disease and death in men and women of the Scottish Heart Health Study: cohort study. *BMJ* 1997; 315: 722-729.

11. Ding P, VanderWeele TJ. Sensitivity Analysis Without Assumptions. *Epidemiology* 2016; 27: 368-377.

12. VanderWeele TJ, Ding P. Sensitivity Analysis in Observational Research: Introducing the E-Value. *Ann Intern Med* 2017; 167: 268-274.
